# The Structural Basis of *Mycobacterium tuberculosis* RpoB Drug-Resistant Clinical Mutations on Rifampicin Drug Binding

**DOI:** 10.3390/molecules27030885

**Published:** 2022-01-28

**Authors:** Arnold Amusengeri, Asifullah Khan, Özlem Tastan Bishop

**Affiliations:** 1 Research Unit in Bioinformatics (RUBi), Department of Biochemistry and Microbiology, Rhodes University, Grahamstown 6140, South Africa; ahnoamu@gmail.com; 2 Department of Biochemistry, Abdul Wali Khan University Mardan (AWKUM), Mardan 23200, Pakistan

**Keywords:** drug resistance, mutations, rifampicin, *rpoB*, molecular dynamics simulations, dynamic residue network analysis

## Abstract

Tuberculosis (TB), caused by the *Mycobacterium tuberculosis* infection, continues to be a leading cause of morbidity and mortality in developing countries. Resistance to the first-line anti-TB drugs, isoniazid (INH) and rifampicin (RIF), is a major drawback to effective TB treatment. Genetic mutations in the β-subunit of the DNA-directed RNA polymerase (*rpoB*) are reported to be a major reason of RIF resistance. However, the structural basis and mechanisms of these resistant mutations are insufficiently understood. In the present study, thirty drug-resistant mutants of *rpoB* were initially modeled and screened against RIF via a comparative molecular docking analysis with the wild-type (WT) model. These analyses prioritized six mutants (Asp441Val, Ser456Trp, Ser456Gln, Arg454Gln, His451Gly, and His451Pro) that showed adverse binding affinities, molecular interactions, and RIF binding hinderance properties, with respect to the WT. These mutant models were subsequently analyzed by molecular dynamics (MD) simulations. One-hundred nanosecond all-atom MD simulations, binding free energy calculations, and a dynamic residue network analysis (DRN) were employed to exhaustively assess the impact of mutations on RIF binding dynamics. Considering the global structural motions and protein–ligand binding affinities, the Asp441Val, Ser456Gln, and His454Pro mutations generally yielded detrimental effects on RIF binding. Locally, we found that the electrostatic contributions to binding, particularly by Arg454 and Glu487, might be adjusted to counteract resistance. The DRN analysis revealed that all mutations mostly distorted the communication values of the critical hubs and may, therefore, confer conformational changes in rpoB to perturb RIF binding. In principle, the approach combined fundamental molecular modeling tools for robust “global” and “local” level analyses of structural dynamics, making it well suited for investigating other similar drug resistance cases.

## 1. Introduction

Tuberculosis (TB) remains a major public health problem globally, with resistance to rifampicin (RIF) and isoniazid (INH), the main anti-TB first-line drugs, varying according to geographical location [1,2]. The number of TB patients diagnosed and treated for multidrug-resistant tuberculosis (MDR-TB) is increasing worldwide, and treatment success rates in patients with drug-resistant TB remain unacceptably low [1]. According to the World Health Organization (WHO), 9.0–11.1 million new cases and 1.1–1.3 million TB-related deaths were estimated in 2018 [1]. The global health problem due to tuberculosis has worsened with the increasing emergence of *Mycobacterium tuberculosis* complex strains that are resistant to RIF and INH. As recommended by the WHO, the timely detection of drug resistance is essential for the appropriate treatment of patients with tuberculosis and for limiting the further spread of MDR-TB [1,3]. The drug-resistant form of TB originates from the development of different mutations [4,5,6]. Drug resistance poses an immense threat and presents new challenges in diagnostics, surveillance, and therapeutic methods employed in treating TB. Reports of increasing drug-resistant TB cases have emphasized the need for research to understand the basic mechanism of drug resistance [7,8,9].

**Figure 1:**
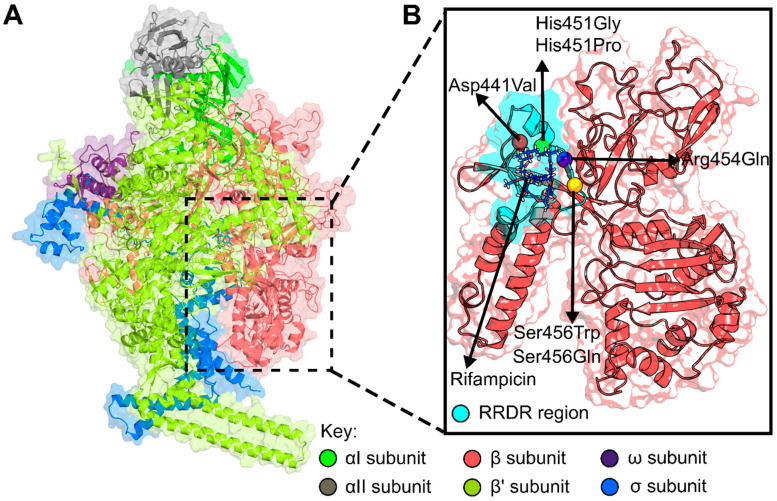
(**A**) Structural representation of the multiunit *M. Tuberculosis* RNAP catalytic core complex (consisting of α_I_, α_II_, β, β′, ω subunits) bound to an σ initiation factor to form a holoenzyme, PDB ID: 5UHC [33,34]. (**B**) Focused image of mutations under investigation (shown as spheres) within the rpoB model (residues 160 to 600). The RRDR region is mapped on the structure and colored cyan. RIF is shown as sticks and colored blue.

RIF, a member of the rifamycin family of antibiotics, is a critical component of the combination therapy regimen used to treat active TB [10]. Since its approval for clinical use in late 1960s, RIF has made its mark as a core therapy for infections sustained by various bacteria, including TB (*Mycobacteria tuberculosis*), osteomyelitis (*Staphylococcus aureus*), meningococcal disease (*Neisseria meningitidis*), leprosy (*Mycobacterium leprae*), and gonorrhea (*Neisseria gonorrhoeae*) [11]. Regarding *M. tuberculosis*, RIF exerts its effect by inhibiting DNA-dependent RNA polymerase (RNAP) [12]. RNAP is responsible for transcriptions and gene expressions in all living organisms. Bacterial RNAP is a multi-subunit complex consisting of five subunits (α_2_ββ′ω) (Figure 1A). The two largest RNAP subunits, β and β′, in coordination with Mg^2+^ ions, form the active center for the catalysis of nucleotide polymerization [13,14]. RIF binds to an adjacent pocket in the β-subunit encoded by the *rpoB* gene (Figure S1) [15,16], sterically inhibiting the extension of nascent RNA, and eventually blocking the bacterial proliferation [12].

It is estimated that 484,000 TB cases reported worldwide in 2018 were RIF-resistant [1]. More than 95% of the *M. tuberculosis* clinical strains resistant to RIF harbor mutations in the 81 base pair region of the *rpoB*, known as the RIF resistance-determining region (RRDR) (Figure 1B). The region spanning codons 433 to 458 (corresponding to 507 to 533 in the *Escherichia coli* numbering system, respectively) [7,17,18,19], which is a hotspot region, mainly consists of single amino acid substitutions [20]. The substitutions at codons 441, 451, and 456 (corresponding to codons 516, 526, and 531 of the *E. coli* coordinates) occur frequently and have often been used as excellent markers for the detection of resistance in RIF-resistant *M. tuberculosis* isolates [7,21,22,23]. Mutations outside the RRDR region in *rpoB* have also been reported in association with RIF resistance, but rarely [23,24,25]. Since mutations are considered critical factors associated with the acquisition of drug resistance, the elucidation of the mechanism(s) of action of the clinically important *rpoB* gene mutations that cause RIF resistance have become increasingly important in the development of novel anti-TB therapeutics. In this study, we seek to understand the structural implications of resistance-conferring mutations, occurring in the rpoB subunit, on RIF binding.

Previous computational studies have illustrated the structural consequences of amino acid variations in the RNA polymerase protein on drug binding affinities and drug-target stabilities [19,26,27,28]. The deterioration in electrostatic interactions and the rearrangement of the binding pocket to alter its shape complementarity, leading to poor binding affinities for mutations associated with positions 456 and 441 (Ser456Leu and Asp441Val), have been cited [15,19]. Likewise, substitutions at position 451 (His451Ser/Met/Glut/Asp/Tyr/Arg) induced detrimental effects on protein–ligand stability, as well as affinity [27,28]. While these studies have extensively explored thermodynamic aspects of RIF binding and have captured relevant biophysical interactions, less attention has been paid to conformation selection and intraprotein communication regulation. For instance, it is not clear whether residues within the RRDR region are key intermediaries in the intraprotein information flow, nor is it clear how mutations within this hotspot region influence the protein conformation freedom of the free energy landscape, ultimately dictating the drug binding kinetics. The systematic approach of combining molecular modeling and network analysis techniques has previously provided a versatile strategy of elucidating drug resistance mechanisms, and to a greater scope, the mechanistic ramifications of the mutations to drug binding [29,30,31]. Here, we screen non-synonymous single nucleotide polymorphisms (SNPs) reported in the mutation bioinformatics identification (MUBII-TB-DB) database [32], in order to unveil their influence on RIF binding in mycobacterial RNAP. We employ molecular docking, molecular dynamics (MD) simulations, and dynamic residue network (DRN) analysis techniques to prioritize the database mutations based on their impact toward RIF binding affinity. Here, the mutant-ligand stability, conformation selection, binding affinity, and intraprotein communications are inferred with respect to the bound wild-type (WT) model to understand the structural insight and underlying mechanisms of RIF resistance.

## 2. Results

### 2.1. Rifampicin Resistant-Associated Mutations Were Prioritized

The MUBII-TB-DB database held 133 RIF-rpoB drug-resistant mutations cataloged from the published literature (Supplementary_file S1, Supplementary_file S2) during the planning of this study (as of 6th September 2018). The most common RIF-resistant mutations in *M. tuberculosis* are reported in the RRDR region and are listed in the MUBII-TB-DB according to the *E. coli* genome coordinate position. Some *M. tuberculosis* strains were found to carry multiple point mutations. Therefore, the target region sequences were initially aligned to the H37Rv reference strain, as well as *E. coli*, using ClustalW in MEGA7 [35], in order to ascertain the position of target mutations [36,37]. Notably, the molecular interaction patterns governing RIF resistance and sensitivity for most mutations have previously been published in different in silico, and experimentally resolved, RIF-bound rpoB crystallographic structural assessments [38,39]. For this reason, we prioritized 30 mutations deposited to MUBII-TB-DB 5 years before the planning of the study, whose molecular insights, with respect to RIF resistance, have barely/not been assessed so far. The majority of these mutations were in the RRDR region of *rpoB*. Mutations occurring outside the hotspot region have been reported to cause an abrupt change in the rpoB structure and have been reported to influence the RIF binding efficiency [40]. About 30% of MDR-TB presented RIF-resistant mutations located outside the RRDR region [40].

The post-docking analysis revealed varied binding patterns of RIF with the WT as well as the mutated models in terms of docking scores, the formation of hydrogen bonds, and their associated distances.

**Figure 2:**
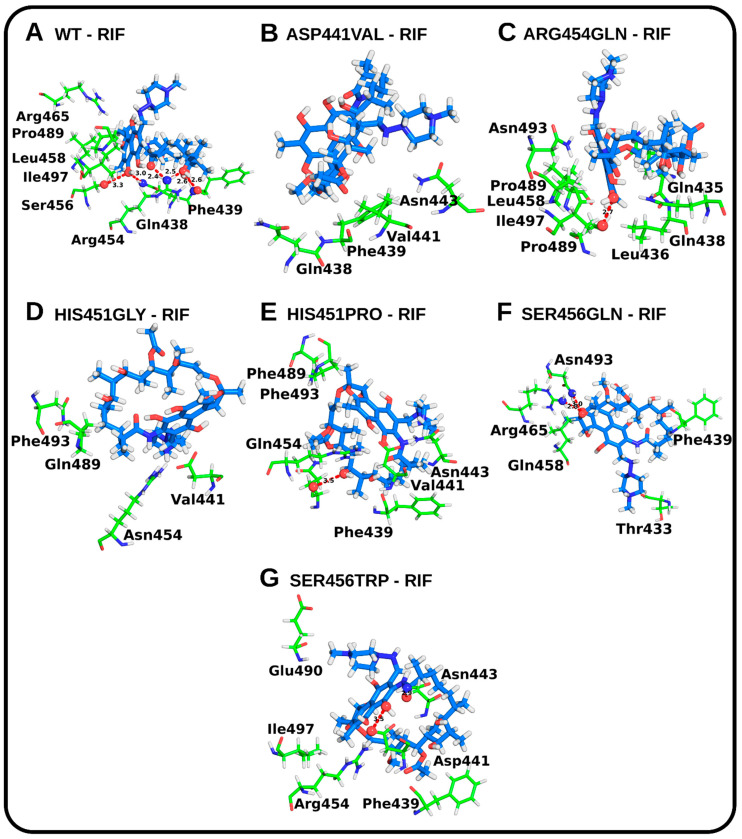
Post-docking analysis: The three-dimensional illustration of molecular interactions of RIF with the WT and mutated forms of *M. tuberculosis* rpoB. RIF is shown as blue sticks colored by heteroatom. Interacting atoms and associated residues are shown as spheres and sticks (colored green), respectively. Hydrogen bonds are shown as dashed red lines.

Generally, the mutated complexes yielded higher binding free energy values relative to the WT rpoB-RIF complex (–13.8 kcal/mol), indicating that the mutations, regardless of their positions within the RIF binding pocket, reasonably abolish protein–drug binding (Table S1). Few mutations were found to completely abolish RIF binding, with substantially higher ligand root-mean-square deviation (RMSD) scores (ligands in the mutant model compared to ligands in the WT model) and poor docking scores. The intermolecular interactions were also investigated. The results evidently indicated that the prime difference in the binding affinity could be due to differences in binding pocket residues. The RIF-mutant interacting residues were found to be largely different from those of the native complex and, thus, could be responsible for the comparatively poor docking scores (Figure 2 and Figure S2).

In the case of the WT rpoB protein, the RIF binding site residues, i.e., Arg454 and Phe439, formed hydrogen bonds with RIF. An amino group of Arg454 formed two hydrogen bonds with a carbonyl oxygen and hydroxyl (-OH) side chain, whereas the amide nitrogen of Phe439 formed one conventional hydrogen bond with an oxygen acceptor atom. The Asp441Val, His451Gly, His451Pro, Arg454Gln, Ser456Gln, and Ser456Trp models were found as the top mutants with respect to RIF binding hindrance and their poor ligand docking scores of −6.1 kcal/mol, −5.0 kcal/mol, −6.5 kcal/mol, −6.1 kcal/mol, −6.0 kcal/mol, and −5.4 kcal/mol, respectively, compared to the WT complex (–13.7955 kcal/mol). The visual inspection of the docked poses of the mutated rpoB complexes revealed that RIF either developed few hydrogen bond interactions with the binding sites residues or abolished them altogether (Figure 2). Likewise, high ligand RMSD scores were yielded during the superimposition of the mutant models against the WT complex (Table S1).

### 2.2. Mutations Variably Influenced the Protein and Ligand Stability

**Figure 3:**
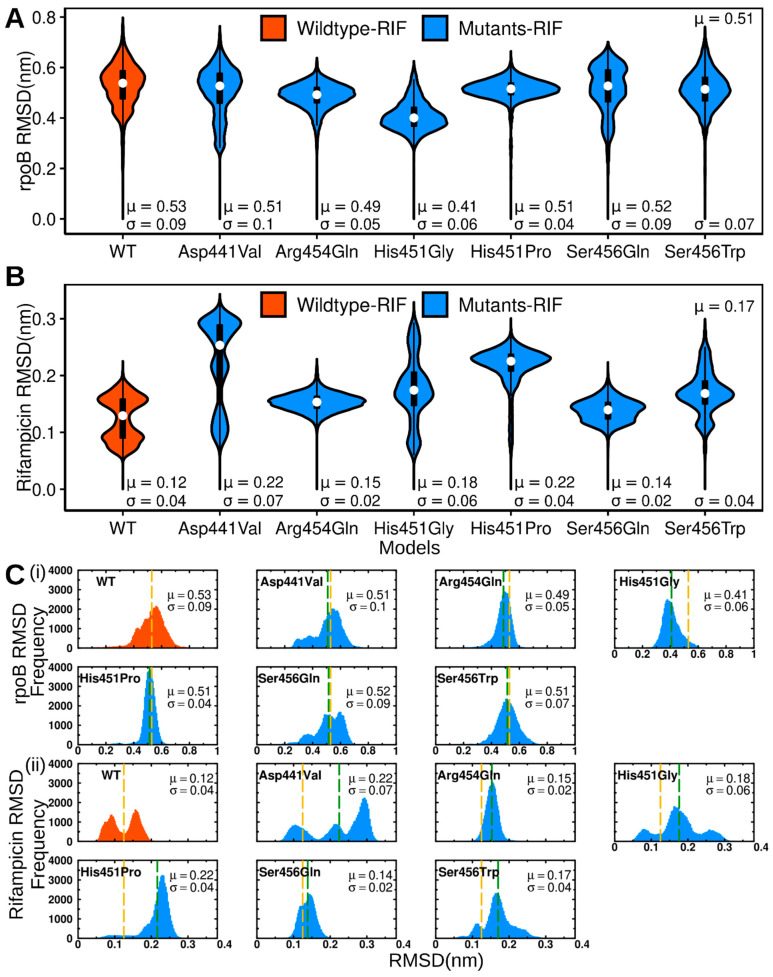
Violin plots illustrating the kernel density distribution frequency of (**A**): Protein RMSD, (**B**): Ligand RMSD. Color key: Red: bound WT, Blue: bound mutants. Embedded boxplots highlight the data distribution (25th (bottom) and 75th (top) percentiles). The white dot indicates the median value. (**C**) (**i**) and (**ii**). Histogram representation of RMSD distribution for (**i**) the protein, and (**ii**) RIF. Color key: red: bound WT, blue: bound mutants. The mean RMSD value is highlighted as dashed lines: yellow: representing the WT, and green: representing mutants.

Six out of the thirty mutated rpoB models were identified as the top mutants possessing unfavorable protein–ligand interactions. To elucidate the impact of mutations on the dynamic behavior of ligand binding, 100ns MD simulation experiments were performed on the WT and the six mutated models in the complex with RIF. Duplicate runs were carried out for the WT (see Figures S3 and S4). MD simulations have been applied in various studies to exhaustively highlight structural mechanisms for differences in ligand bindings between the native protein and mutated targets [19,26,27,28,30,31]. Despite experimental evidence showing that RIF effectively binds to the WT rpoB protein at a pH of 7.4 based on physiological conditions [41,42], it remains unclear what the binding state of the drug and its associated conditions are there with the RIF-resistant mutants. Mutations are known to stimulate resistance by associating with the drugs at distinct pH levels [43,44,45,46]. It should be noted that the results discussed here illustrate the binding behavior of RIF in its neutral state, compared to the WT and the mutated proteins. To examine the general conformational divergence from the baseline structure (at zero nanoseconds), the protein and ligand RMSDs were computed. Calculations of the protein RMSD were based on the backbone atom positions. Initially, based on the RMSDs, it was found that both WT runs were in equilibrium. Moreover, the extent of divergence was small; therefore, dependable conclusions could be drawn from both runs: the models acquired comparatively similar conformational spaces considering the fair agreement in kernel density estimation plots, and the dimension projections of conformational shifts on the free energy landscape were lower (Figure S3). Protein RMSDs of both WT and the mutated models plateaued early to values in the range of 0.4 to 0.6 nm, on average; this suggests that the overall structural folds were fairly maintained (Figure S4). The average RMSD values acquired were below 0.53 nm, while the distribution median (interquartile range) recorded, in general, was 0.50 (0.09) nm (Table S2, Figure 3A,C(i)). The WT, Asp441Val, and Ser456Gln models displayed multimodal RMSD distribution patterns and the largest standard deviation (*sd*) values of 0.08 (on average), 0.1, and 0.09, respectively. This indicated that the systems were in equilibrium between multiple conformers. Conversely, the Arg454Gln, His451Gly, His451Pro, and Ser456Trp models showed normal RMSD distribution density curves and recorded the lowest *sd* values: 0.05, 0.06, 0.04, and 0.07, respectively. This suggests that a single, more stable equilibrium state was sampled.

The conformation adaptation of RIF during the simulation was assessed by measuring its RMSD. The ligands displayed system-specific conformation variations; RIF acquired normal (Arg456Gln and His451Pro), bimodal (WT (run 1 and run 2)), Ser456Gln, Ser456Trp), and trimodal (Asp441Val, His451Gly) RMSD frequency distribution density curves (Figure 3B,C(ii) and Figure S3A).

The latter possessed the largest *sd* values (Asp441Val: 0.07 and His451Gly: 0.06, respectively) and exhibited a large RMSD variability. This suggests that the associated mutations largely destabilized the RIF’s binding pose (Figure 3B and Figure S5). Taken together, (I) the WT protein accessed alternative conformers, possibly due to dual binding orientations acquired by RIF during simulation; and (II) mutated models displayed mixed (smaller and larger) conformation proportions with respect to the WT, indicating that the changes in binding site architecture can variably influence both protein and ligand stabilities.

Structural fluctuation at the residue level was monitored using root-mean-square fluctuation (RMSF) calculations, which highlight the per residue mobility in relation to its average position. Looking at the general layout of RMSF profiles, minimal differences were observed between the WT and mutated models: overall, residues 284–286, 397–418, and 595–600 were recorded with the largest fluctuation values (>0.34 nm, on average) (Figures S6 and S7). All the mutated models exhibited lower RMSF values compared to the WT, suggesting that the mutations favor the systemic relaxation.

**Figure 4:**
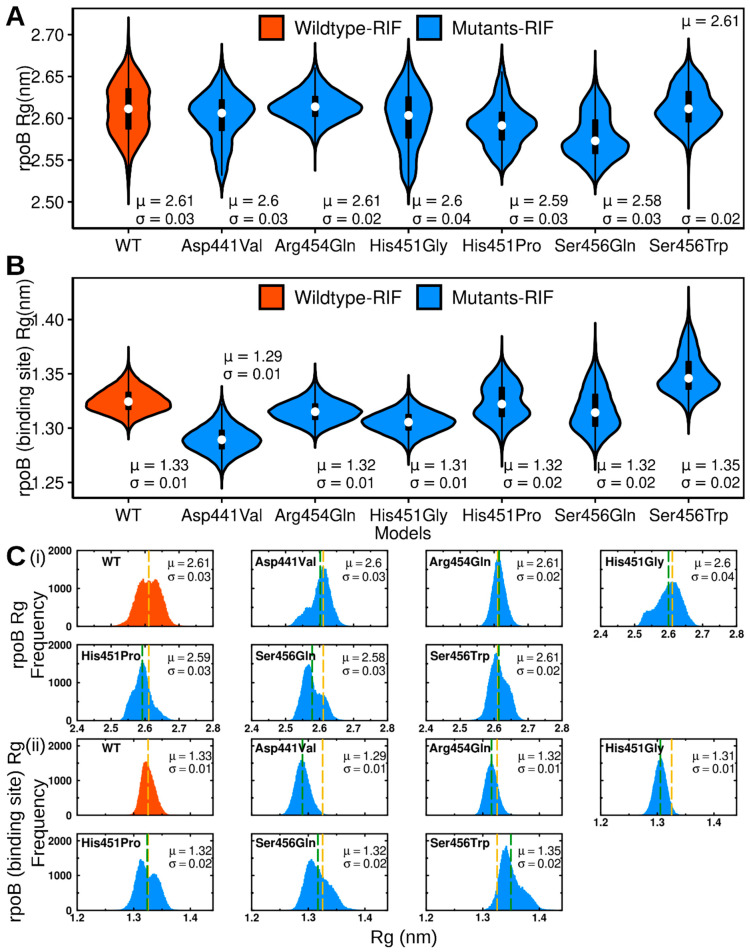
Violin plots illustrating the kernel density distribution frequency of the radius of gyration for (**A**): The entire protein structure, (**B**): residues within the RIF binding site. Color key: red: bound WT, blue: bound mutants. The boxplots within highlight the data distribution (25th (bottom) and 75th (top) percentiles). The white dot indicates the median value. (**C**) Histogram representation of *Rg* distribution for (**i**) the entire protein and (**ii**) RIF binding site. Color key: red: bound WT, blue: bound mutants. Dashed lines represent the mean *Rg* value: yellow: bound WT, green: bound mutants.

The radius of gyration is a measure of compactness, with respect to the proteins’ center of mass. Large *Rg* values correspond to a less tight packing of atoms, and vice versa. All models acquired average *Rg* values in the range of 2.58 to 2.61 nm, suggesting that minimal changes in protein compaction occurred in mutated models, with respect to the WT (Figure 4A,C(i), Figure S3B and S8). Based on *Rg* variability, the His451Gly (0.04 nm), WT, Asp441Val, His451Pro, Ser456Gln (0.03 nm each), Arg454Gln, and Ser456Trp (0.02 nm each) were found with large-to-small *sd* values, respectively. To investigate the local effects of the mutations on the packing density of residues within the RIF binding site, *Rg* calculations were performed on residues 166–181, 374–389, 430–440, 449–469, 473–475, and 488–501 (Figure 4B,C(ii) and Figure S3B). His451Pro, Ser456Gln, and Se456Trp exhibited larger *Rg* variations (*sd* values of 0.02 each) relative to WT, Asp441Val, Arg454Gln, and His451Gly. These results suggest that: (I) Asp441Val confers insignificant effects on the protein packing density, both globally and locally (considering the RIF binding region). (II) His451Pro and Ser456Gln remain mainly intact, except for looser packing in the rifampicin binding region. (III) The His451Gly and Arg454Gln mutations majorly influence (increase and decrease, respectively) the spatial packing of the entire structure. (IV) While structural compaction is enhanced globally in Ser456Trp, structural expansion is favored in the RIF binding region. The above results clearly show that mutations can variably influence conformational compaction/expansion and, likewise, alter the shape of the complementarity aspects with respect to the ligand binding site.

### 2.3. Mutations Favor Conformational Rigidity

**Figure 5:**
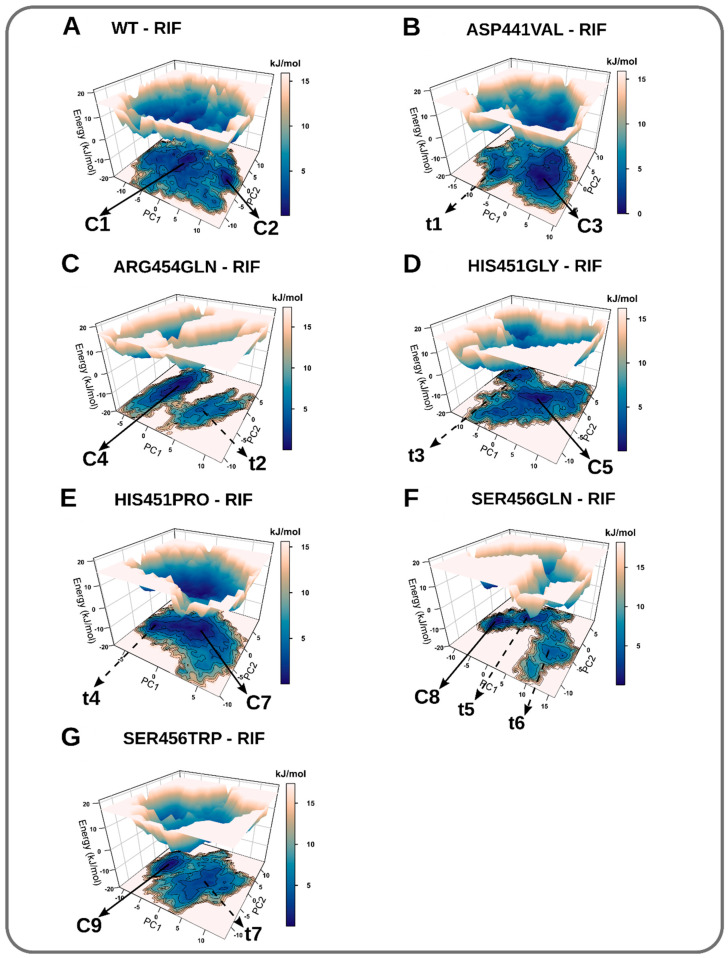
Protein free energy landscapes computed as a function of PC1 and PC2 for WT and mutated systems. Color ranges from white (energy maxima) to blue (energy minima). Labels: c: conformers, t: transition substates.

The investigation of conformation redistribution, due to mutation/ligand binding, is important for understanding resistance mechanisms. Essential dynamics were carried out in order to examine how the single point mutations reorganize the conformation populations in a reduced dimensional subspace. Often, principal components are sorted in such a way that the top few retain the most variation; the first two (PC1 and PC2) typically describe dominant systemic motions [47]. In general, PC1 and PC2 (from a total of 3659 eigenvectors) captured more than 40% of the total variance (Table S3). Two-dimensional projections suggested that the intrinsic motions were highly restricted, and the conformational subspaces were relatively limited (Figure S9). Relative to the WT, most mutants covered a restricted spatial area, suggesting an overall decrease in the accessible degrees-of-freedom. The projections of the proteins’ free energy landscape (FEL), as a function of significant (slow) modes, provided a robust representation of dominating conformation ensembles and allowed a clear visual interpretation of the folding pathways. The intermediate basins (labelled “t”) were best described as transitional conformations equivalent to metastable states that lead to the bottom of the folding funnel (the native state, labelled “C”) (Figure 5) [48,49]. The WT explores two global-wide energy minimas (C1 and C2) separated by low lying energy barriers. This suggests that rpoB natively adopts two conformations during the RIF binding, as previously highlighted in RMSD calculations.

All mutants adopted single global free energy minima; however, they were trapped transiently in local energy minima wells towards the bottom of the funnel. Asp441Val, Arg454Gln, His451Gly, His451Pro, and Ser456Trp each sampled a single metastable substate (t1, t2, t3, t4, and t7, respectively), whereas Ser456Gln visited two metastable substates (t5 and t6). These observations agree with the RMSD kernel density estimations plots (see Figure 3), which appropriately highlighted that Ser456Gln acquired large conformational variability. Lower trace values were observed for all the mutants (when compared to the WT), suggesting that the concerted atomic displacements yielded, due to mutations, were generally reduced (Table S3). Thus, the mutations confer structural rigidity. The projections of concerted motions using porcupine plots indicated that the major motions in the mutated models were dominated by the distant loop region (outside the RIF binding site) between residues 397–418. This region also exhibited large residual fluctuations, as described in the RMSF calculations (Figure S10). Globally, the WT model displayed larger atomic motions in general, whereas the mutated models showed reduced motions. The comparisons of the structures occupying dominant energy wells (conformers of the WT, versus the mutants) revealed large structural displacements across all mutants in the entire model following the superposition with the WT (Figure S11). Relative to the WT, mutated models recorded RMSD values in the range of 0.801 to 0.904 nm, emphasizing that the mutations induced considerable structural rearrangements (Table S4). In summary, mutations generally discouraged large atomic shifts relative to the WT, besides sizably restricting the conformational freedom of rpoB during the RIF binding. These observations could be considered a linchpin in the impairment of drug recognition in all mutants.

### 2.4. The Impact of Mutations on Protein-Ligand Binding Affinity

Intermolecular hydrogen bond numbers (H-bonds) play a key role in stabilizing protein–ligand complexes. Compared to the WT (three H-bonds), Arg454Gln (four H-bonds), and Ser456Trp (four H-bonds), which generally recorded higher intermolecular hydrogen numbers, Asp441Val (two H-bonds), His451Gly (two H-bonds), His451Pro (two H-bonds), and Ser456Gln (one H-bond) yielded lower numbers, on average (Figure S12). These results suggest that the latter’s mutations largely destabilized the RIF–rpoB interactions. Protein–ligand interactions were examined by considering representative structures occupying energy minimas (dominant conformers) (Figure S13). Comparable to the WT, Arg454Gln, Ser456Trp, and Arg454Gln generally formed higher (four or more) conventional hydrogen bonds with RIF. The WT residues Arg465, Ser456, Gln435, Gln438, and Phe439 formed conventional hydrogen bonds with RIF. Notably, interactions with residue Phe439 were present across all mutations, except His451Gly and His451Pro. Both models (His451Gly and His451Pro) displayed diminished molecular interactions in general, suggesting that the mutations at position 451 conferred large detrimental effects on binding contacts.

Single point mutations occurring at ligand binding sites have been shown, in the past, to influence the binding thermodynamics of ligands [30,50]. Binding free energy (BFE) calculations provide a quantitative overview of drug–receptor affinities. MM/PBSA functions have been employed in previous works to estimate the total relative binding free energy, as well as the contribution of individual residues to binding through the per residue decomposition method, with promising accuracy [30,51,52,53]. Table 1 displays a summary of the results obtained. In general, the van der Waals interaction (*ΔE_vdW_*) is the driving force for the RIF–rpoB interaction, whereas polar energy (*ΔG_polar_*) is detrimental to binding. Relative to the WT (approximately −128.6 kJ/mol on average), RIF bound, with an increased affinity, to Arg454Gln (approximately −213.4 kJ/mol), His451Gly (approximately −138.2 kJ/mol), and Ser456Trp (approximately −216.3 kJ/mol) mutants, while its binding to Asp441Val (approximately −25.0 kJ/mol), His451Pro (approximately −90.2 kJ/mol), and Ser456Gln (approximately −71.5 kJ/mol) was not favored. While the computed total binding free energy values were expected to be nearly similar, with respect to the duplicate runs of the same system, the WT run 1 and run 2 recorded large variations. The electrostatic energy term (*ΔE_elec_*) was the predominant factor dictating the observed fluctuations. This was not only evident within the duplicate runs of the WT protein, but also across the mutants. It should be pointed out that resultant binding free energy values are as accurate as the scoring functions. Additionally, MMPBSA computed the relative binding free energy, which does not equal the real binding free energy, since we did not estimate the entropy contribution to binding. Despite these shortcomings, both WT runs showed remarkable consistencies in their per residue energy decomposition profiles (see Figure S14), giving us confidence in the obtained results. The residue decomposition of the BFE revealed three key residues in the WT model: Leu458, Pro489, and Ile497, contributing substantially to favorable ligand binding (Figure S14, Table S5). Interestingly, mutations arbitrarily disrupted the per residue thermodynamic profiles across all mutated models (Figure S14). Moreover, it is likely that the mutations induced far-reaching allosteric effects that strongly influenced the thermodynamic balance of binding. As such, the models yielded mutant-specific patterns of residues that contributed substantially to the total BFE (Figure S14, Table S5). The interactions with residue 454 (Arg454) did not favor ligand binding across all models (including WT), except the Asp441Val and Arg454Gln mutants. Markedly, the interactions with Glu487 that were identified as detrimental to binding in the WT system, contrarily, yielded favorable binding energy values in all mutants except Asp441Val.

Previous computational studies on systems with mutations at positions 456 and 441 (i.e., Ser456Leu and Asp441Val) yielded concordant results with those of Ser456Gln and Asp441Val, considering the total binding free energy. Relative to the WT, Ser456Leu and Asp441Val exhibited a decrease in their total binding affinity in that order, which could be attributed to weakened electrostatic interactions [19]. Particularly, the weakened electrostatic interactions with Arg454 were highlighted as key to the development of resistance. Zhang et al. reported weakened binding affinities for complexes with various residue substitutions at position 451 (His451Asp, His451Tyr, H451Arg) [28]. However, our models yielded mixed results for this position. His451Gly (−138.2 kJ/mol) and His451Pro (−90.2 kJ/mol) were recorded with increased and decreased binding affinities, respectively. Taken together, the Arg454Gln and Ser456Trp mutations leveraged the drug–receptor complex stability, as demonstrated by higher hydrogen bond numbers larger binding affinity values, while the Asp441Val, His451Pro, and Ser456Gln mutations generally did not favored the drug binding and interactions.

### 2.5. Mutations Conferred Common Disparaging Effects on Critical Communication Hubs

Graphs of the residue interaction network have been widely employed in the identification of key amino acids involved in the regulation of protein functions [30,52,53,54]. Analyses of network graphs were conducted based on the average shortest path (*L*) and betweenness centrality (*BC*) metrics. When computed over a trajectory, the average *L* highlights how easily accessible a residue is for communication. In principle, regions with low average *L* values are better disposed to transmit information. Generally, both the WT and mutated models exhibited minimal differences in regard to residues possessing low average *L* values. The 1X*sd* from the mean value was used as a threshold to identify low average *L* regions (dips) (Figure S15). Considering the WT model (native representation), the residues 177–188, 203–208, 213–214, 309–320, 338, 342, 369–385, 437, 455–458, 463, 474–484, 499, 505–514, and 532–535 yielded low average *L* values in general, signifying highly accessible communication hubs. Relative to the WT, all mutated models were found with lower threshold values, indicating an overall decrease in average shortest path lengths (Figure S15).

**Figure 6:**
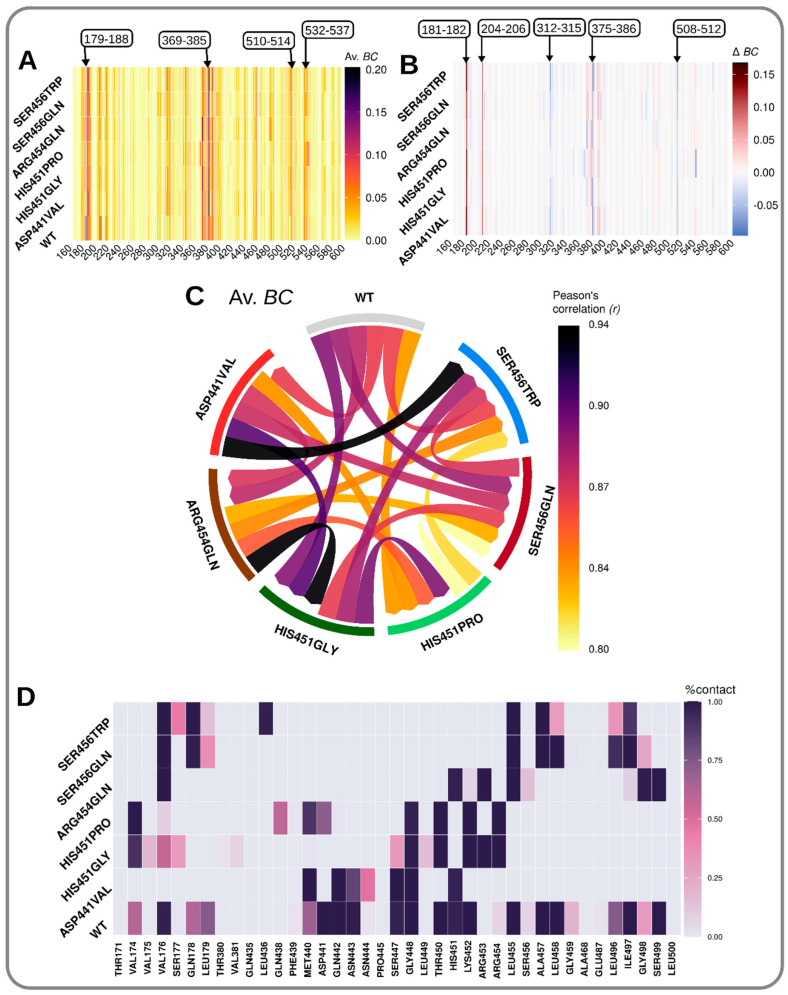
Analysis of average *BC* results: (**A**) Per residue average *BC* values illustrated using a heatmap. (**B**) The residue changes in average *BC* index (*ΔBC*) because of mutations (WT model and less mutated model). (**C**) Pairwise Pearson’s correlation among models with respect to average *BC* profiles: comparison of linear relationships between the WT and the six mutated models in this dataset. The color ranges from yellow: the smallest correlation value, to black: the largest correlation value. (**D**) Per residue weighted contact heatmap. Color range is from white (0% frequency of occurrence) to violet (100% frequency of occurrence). Per residue contact network map representation is shown in Figure S17.

*BC* provides a hierarchical arrangement of highly influential residues in a social network by considering their ratios of participation in all pairs of the shortest paths. Residues possessing high average *BC* indexes are associated with signal propagation and are deemed critical intermediaries for intraprotein communication. Generally, both WT and the mutated models displayed largely similar average *BC* profiles and were strongly correlated, as exhibited by high pairwise Pearson’s correlation values (Table S6, Figure 6A,C and Figure S16). The 2X*sd* value was used to identify residues that yielded large average *BC* values (peaks) with respect to the WT model (the native representation). Generally, most residues that yielded high *BC* values were located within the ligand binding region (for instance, residues 179–182, 374–380, 384–387, and 456), indicating their importance in achieving native protein–drug interactions (Table S7). Among these, the residue 456 was placed within the RRDR [23,55,56], suggesting that the residue plays a key role in achieving standard drug potency. Moreover, residue 376, located outside the RRDR region, has previously been shown to confer drug resistance upon its mutation [23]. Interestingly, changes in average *BC* values, due to mutations, mostly occurred in these signaling hubs across all mutated models in general, including residues 181–182, 204–206, 375–380, 384–386, and 512, as shown in Figure 6B and Table S8. This is indicative of the common derogatory effects of mutations on critical communication hubs and could serve as molecular signatures distinguishing drug-susceptible WTs from drug-resistant mutants [30]. Residues unique to each model, with respect to our dataset, were also identified, including: 179, 379 (Asp441Val); 370, 379, 477, and 478 (Ser456Trp); 365, 366, 510, 539, and 540 (Arg454Gln); and 387, 390, 187, 209, 371, 388, 391, 392, and 461 (His451Gly) (Table S8). These residues might be useful in explaining the differences in phenotypic characteristics, or, perhaps, the severity of resistance exhibited by each mutant. Despite the pairwise Pearson’s correlation showing the strong correlated arrangement of the network layout among the models (*r* range: 0.80–0.94) with respect to the average *BC* (Figure 6C, Table S6), the comparisons suggested that the mutated models might be distant from the WT in the order of His451Pro > Ser456Trp > Asp441Val > Arg454Gln > Ser456Gln > His451Gly > WT.

In summary, the mutations investigated here altered the native information flow network, characterizing the drug-susceptible WT system by increasing and decreasing the overall reachability and connectivity, respectively, of most residues charged with adjudicating intraprotein communications in their bound state. While these residues may not be directly associated with the RRDR region, their proximity to this area, as well as residues within the RIF binding pocket, could explain the associated detrimental impact of mutations on ligand binding.

Residue contact maps have been used in the past to analyze differences in residue interactions and as a tool for general protein structure comparisons [57,58]. We employed contact maps to scan through the trajectories and to identify shared and unique contacts between the WT and mutated models. Pairwise comparisons of the WT and mutants were carried out with respect to the mutated residue positions. Generally, the WT and all mutants were glued by numerous mutual molecular contacts (Figure 6, Figure S17). We hypothesized that the differential contacts, due to mutations, could be responsible for modulating the local thermodynamic changes and, hence, RIF stability. Specifically, we focused our analysis on the lost/emerging contacts that contributed substantially to the total binding free energy. Compared to the contacts exhibited in the WT model, residues Thr450 (Asp441Val), Gln438 (Arg454Gln), Thr380, Val381, Leu455; Ser177, Val175 (Ser456Gln), Gln435 (Ser456Trp), Phe439, and Gln442 (His451Pro) either emerged or lost contacts due to their associated mutations. Among these, Gln438, Gln435, Leu436, and Phe439 directly contributed to the total binding free energy in the WT (See Table S5). Other residues, including Thr450, Leu455, Val175, and Ser177, as well as the neighbor residues His451, Arg454, Arg173, and Val176, were also charged with maintaining protein–ligand affinities. Based on the per residue binding free energy contribution values, these interactions might be categorized either as stabilizing (associated with positive energy values) or destabilizing (associated negative energy values).

In summary, the above results provide evidence that the mutations confer resistance by losing/making novel contacts, particularly with residues participating in protein–ligand binding. While how the degree of association (weights) contributes to inefficiency in binding may not yet be established, it will certainly be exciting to investigate the consequences of fine-tuning these associations in order to counteract resistance.

## 3. Materials and Methods

### 3.1. Data Retrieval

#### 3.1.1. Protein Preparation

The crystal structure of the DNA-directed rpoB chain was retrieved from the protein data bank (PDB) under the ID, 5UHC [33]. Before docking, the structure was checked for missing atoms, bonds, and contacts. The energy of the retrieved protein molecule was minimized after 3D-protonation using the default parameters of the MOE energy minimization algorithm (gradient: 0.05, force field: MMFF94X) [59].

#### 3.1.2. Ligand Preparation

The ligand RIF used in this study against rpoB was retrieved from the crystal structure of the complex rpoB protein. The ligand was energy minimized using the default parameters of the MOE energy minimization algorithm (gradient: 0.05, force field: MMFF94X) [59]. The minimized molecule was saved in the mdb file format as a ligand input file.

#### 3.1.3. The Retrieval of *Mycobacterium tuberculosis rpoB* Clinical Mutations

The MUBII-TB-DB repository holds *M. tuberculosis* mutations at seven loci associated with its resistance to first- and second-line antibiotics [32]. The database includes 133 RIF-*rpoB* drug-resistant mutations cataloged from the published studies (Supplementary_file S1, Supplementary_file S2). The repository holds mutations in the hotspot region (RRDR), as well as outside the hotspot region, within the *rpoB* [60,61]. The drug-resistant mutations, which have previously been evaluated with respect to RIF binding in an experimental layout, as well as via molecular docking and MD simulation approaches, were excluded.

### 3.2. Mutant Model Generation and RMSD Calculation

The 3D structures of rpoB mutant models were constructed using MOE software (MOE-2016). The established rpoB protein structure deposited in PDB under the ID, 5UHC, was used as template. All the mutants were 3D-protonated and energy-minimized using the default parameters of the MOE. The native (WT) structure was superimposed on the various mutant models and the root-mean-square deviation values (RMSD) of each pair (WT against mutant) were calculated using the MOE. The docked complexes were analyzed and the interactions between RIF and WT, as well as mutant proteins, were visualized using PyMOL [62].

### 3.3. Molecular Docking of Wild-Type and Mutant Protein Structures with RIF

Molecular docking simulations of RIF, into the binding pocket of the WT and the mutated rpoB models, were performed using the MOE [59]. The triangle matcher docking algorithm was implemented. All the ions were deleted, and the hydrogen atoms were added to the protein by 3D-protonation using the MOE software. The models were then energy-minimized using the default MOE parameters. The structure of the RIF compound was built in the MOE and was energy-minimized using the MMFF94x force field with a gradient value of 0.05. The inhibitor was re-docked into the RIF binding site of the target enzymes while applying the default parameters of the MOE (placement: triangle matcher, rescoring function: London dG) [59]. Ten conformations were generated for the ligand. The selection of the best docking solutions for further analyses were based on consensus-ranking by considering the following criteria: (I) How solid the affinity of RIF and rpoB was, while looking at the binding free energy values, and (II) how similar the docked conformation of rifampicin in mutants, relative to that in the WT, was by looking at the RMSD deviation values.

### 3.4. All Atom Molecular Dynamics Simulations of the rpoB–RIF Complexes

The top effective mutants were prioritized for downstream MD simulation analyses based on the docking scores, RMSD calculations, and receptor–ligand chemical interactions perturbation. Molecular dynamics simulations were performed using GROMACS 2016.4 [63]. The AMBER03 force field was employed [64]. ACPYPE software was used to assign appropriate atomic partial charges and to generate RIF topologies. ACPYPE combines the general amber force field (GAFF) method and the semi-empirical quantum chemistry program, SQM [65]. Default options were applied. Mg^2+^ ions were excluded from all simulation systems [66]. A cubic simulation box with 2 Å space from the edge of the complex was defined and filled with TIP3 (SPC126) explicit water molecules. System charges were neutralized using a 0.15 M NaCl concentration. The final systems were composed of approximately 200,000 particles. While applying the steepest descent algorithm, the models were energy-minimized up to a gradient limit of ≤1000 kJ/mol/nm. One hundred thousand cycles of NVT equilibration, at 300 K as the initial temperature, and NPT equilibration at 1 bar of pressure, ensued. Temperature and pressure coupling at the 0.1 ps and 2.0 ps time constants were managed using the Berendsen thermostat and the Parrinello–Rahman barostat, respectively. All bonds were constrained using the LINCS solver. Non-bonded interactions were allowed a maximum distance of 1.4 nm. Long-range electrostatic interactions were treated using the particle mesh Ewald method, while the van der Waals interactions were evaluated using the Lennard Jones potential. Finally, 100 ns production runs (with a 2 fs timestep) were performed while applying periodic boundary conditions in all directions. Trajectory snapshots were written out every 2 ps.

### 3.5. Preliminary Trajectory Analysis

Systemic conditions including temperature, potential energy, kinetic energy, and pressure were initially checked to assess the quality of experiments (data not shown). Trajectories were analyzed using the GROMACS built-in utilities. *gmx rms*, *gmx rmsf*, *gmx gyration*, and *gmx hbond* were employed to calculate the RMSD, RMSF, Rg, and intermolecular hydrogen bond numbers, respectively.

### 3.6. Essential Dynamics

The principal component analysis (PCA) is a dimension reduction method that has been widely used to characterize important collective motions of proteins [30,52,53]. A detailed theoretical description of PCA is given by Amadei et al. [67]. The *gmx_covar* module of GROMACS was used to pre-align snapshots from the entire trajectory length and immediately after, generating the covariance matrix (C) associated with the C-α atomic positions. The C is a (3 × 3)m matrix where m is the number of residues considered. The Eigenvalue decomposition of the C-matrix and the pre-sorting from the highest to the lowest variance was carried out using the *gmx_anaeig* tool. Typically, biologically relevant motions can be captured by the combination of top orthogonal modes/principal components (PCs) [68]. Once the functionally relevant motions have been captured, the converged equilibrium probabilities (ΔG(X)) equivalent to the free energy landscape can be expressed as a function of the Boltzmann constant ($K_{B}$) and the absolute temperature (T):

(1) $$\Delta G\left(X\right)=-K_{B}TlnP\left(X\right)$$

where P(X) is the probability distribution of the protein conformations along the PCs.

### 3.7. Binding Free Energy Computations

The effects of mutations on the protein–ligand binding affinity were determined by calculations of binding free energy. The binding free energy computations were carried out on 7500 trajectory snapshots spanning the equilibrated trajectory phase of 85 ns to 100 ns and were sampled at 10 ps time intervals by following the molecular mechanics Poisson–Boltzmann surface area (MM/PBSA) method [69]. The *g_mmpbsa* tool of GROMACS was employed [64]. Briefly, the total binding free energy $\;\Delta G_{binding}$ of a protein–ligand complex can be computed as follows:

(2) $$\Delta G_{binding}=\Delta G_{complex}-\left(\Delta G_{rpoB}-\Delta G_{rifampicin}\right)$$

(3) $$\Delta G_{x}\approx Emm-\left(TS+G_{solv}\right)$$

where $\Delta G_{complex}$, $\Delta G_{rpoB}$, and $\Delta G_{rifampicin}$ denote the Gibbs free energy values of the complex (bound protein), the receptor (free protein), and the ligand, respectively. The *g_mmpbsa* calculates the relative binding free energy; the Gibbs free energy of each component ($\Delta G_{x}$) is equivalent to the sum of molecular mechanical energy (Emm), the solvent accessible area ($G_{solv}$), and the entropic potential (TS). The module utilizes the single trajectory approach, which follows the assumption that the receptor and the ligand endpoint states (bound and free) occupy identical conformations.

### 3.8. Dynamic Residue Network Analysis

The dynamic residue interaction network was performed using MD-TASK [70]. MD-TASK employs the NetworkX algorithm to construct residue interaction network (RIN) graphs. In RIN, amino acid residues represent nodes (in this case, the Cβ atom of amino acids (Cα for Glycine)), and the non-covalent interactions defined by a threshold distance of ≤6.7 Å between residues represents the existing links/edges [71]. The protein RIN was assessed based on the average shortest path (*L*) and the betweenness centrality (*BC*) metrics. *L* highlights the accessibility of a residue for communication. The average shortest path can be constructed using the following equation:

(4) $$L=\underset{i,j\in M}{\sum }\frac{d\left(i,j\right)}{n\left(n-1\right)}$$

where M is the set of nodes (residues) in the protein, and i and j are elements of set M. d(i,j) represents the shortest path from residue i to j, whereas n is the total number of residues in the system. *BC* resolves the degree of connectivity of a residue by looking at its magnitude of participation along the shortest communication paths constructed by all residues, hence its importance as an intermediary for communication. The *BC* index of a residue m in a network can be computed as follows:

(5) $$BC\left(m\right)=\underset{i,j\in M}{\sum }\frac{\sigma (i,j|m)}{\sigma \left(i,j\right)}$$

where *σ*(*i*,*j*) is the total number of the different shortest paths between *i* and *j*, and *σ*(*i*,*j*|*m*) is the count of these *i*-*j* paths that pass through residue m. Calculations were performed on 7500 snapshots sampled at 10 ps intervals spanning the equilibrated last 15 ns of the trajectories. The overall accessibility and connectivity indices were represented by the average value, namely, the average *L* and the average *BC*. In order to make comparisons, the values were initially normalized, as described before [52]. A weighted residue contact map analysis was performed using the MD-TASK webserver (unpublished). Likewise, covalent contacts between a target amino acid and a neighboring residue were considered to be formed if the residue was placed within a radius distance of 6.7 Å or less.

## 4. Conclusions

Tuberculosis is a major health problem that is further exacerbated by the emergence of MDR-TB. Mutations in the chemotherapeutic target, *rpoB*, play important roles in determining the cellular sensitivity and resistance to RIF, the associated first-line anti-TB drug. This work presents a comprehensive examination of molecular mechanisms underlying RIF resistance in selected mutants. At first, six mutated models were prioritized following comparative docking simulations. Relative to the WT, the mutated models Asp441Val, Ser456Trp, Ser456Gln, Arg454Gln, His451Gly, and His451Pro yielded the least favorable protein–ligand binding energies, molecular interactions, and RIF binding pose similarity characteristics. Next, MD simulations, coupled with BFE calculations and DRN, were performed on the seven (six mutants and one WT) protein–ligand complexes to unravel the dynamic effects of mutations on RIF binding. The following testable assertions were demonstrated: (I) Asp441Val and His451Gly largely destabilized the binding orientation of RIF; (II) His451Gly favored structural expansion, whereas Arg454Gln and Ser456Trp favored structural compaction; (III) all mutations induced opposing concerted atomic motions with respect to the WT, while altogether restraining the conformational freedom, where the most significant conformational changes were represented by the motions of loop 397–418; (IV) Asp441Val, Ser456Gln, and His454Pro were detrimental to RIF binding affinity; and (V) all mutations conferred common derogatory effects on critical intraprotein communication hubs, whose impact on ligand binding could be linked to the immediate proximity to the RRDR region and the RIF binding pocket. Lastly, we should consider the contribution of electrostatic interactions for their role in stabilizing protein–ligand affinities and, generally, the molecular interactions with Arg454 and Glu487 might be modified in order to re-sensitize the resistant mutants.

Finally, the experimental verification of the effects of the mutants mentioned here is crucial, and we hope that this study will inspire wet-lab investigation.

## Figures and Tables

**Table 1:** Summary of binding free energy values acquired for each protein–ligand complex.

Systems	*ΔE_vdW_*	*ΔE_elec_*	*ΔG_polar_*	*ΔG_nonpolar_*	*ΔG_binding_* (kJmol^−1^)
WT-RIF run1	−195.7 ± 0.4	−34.6 ± 0.3	140.1 ± 0.2	−20.8 ± 0.0	−111.1 ± 0.5
WT-RIF run2	−218.3 ± 0.3	−62.6 ± 0.3	155.6 ± 0.9	−20.9 ± 0.0	−146.2 ± 0.9
Asp441Val-RIF	−190.1 ± 0.5	77.7 ± 0.5	106.8 ± 1.4	−19.4 ± 0.0	−25.0 ± 1.4
Arg454Gln-RIF	−210.9 ± 0.5	−204.5 ± 1.3	223.5 ± 1.7	−21.5 ± 0.0	−213.4 ± 0.6
His451Gly-RIF	−144.8 ± 0.6	−79.7 ± 0.9	101.7 ± 1.2	−15.4 ± 0.0	−138.2 ± 1.1
His451Pro-RIF	−160.4 ± 0.4	−59.7 ± 0.9	148.3 ± 1.1	−18.5 ± 0.0	−90.2 ± 0.8
Ser456Gln-RIF	−155.0 ± 0.3	11.7 ± 0.6	89.5 ± 0.7	−17.6 ± 0.0	−71.5 ± 0.7
Ser456Trp-RIF	−182.1 ± 0.3	−251.1± 1.2	236.9 ± 1.4	−19.9 ± 0.0	−216.3 ± 0.9

## Data Availability

All datasets generated for this study are included in the article supplementary material.

## Supplementary Materials

The data presented in this study are available in supplementary material. Table S1: Docking scores, RMSD, and post-docking interactions (hydrogen bonds) of rifampicin (RIF) in WT and mutated models of rpoB. Figure S1: A: Graphical representation of the crystal structure of *Mycobacterium tuberculosis* transcription initiation complex, PDB ID: 5UHC [1], containing 3nt RNA in a complex with rifampicin. The structure is superimposed to the wild-type and mutated models. Color code: green: multiunit RNA polymerase complex; brown: 3nt RNA strand; yellow: modeled wild-type structure of the β-subunit; grey: modeled mutated structures of the β-subunit. Rifampicin ligands docked to the structures are shown in sticks. B: 5UHC structure without the RNA strand: Depiction of the rifampicin binding pocket adjacent to the active center [2,3]. C: Zoomed-in image of the rifampicin binding pocket depicting all residues within 3.7 A distance from the ligand atoms. Color code: red: co-crystallized rifampicin bound to 5UHC; blue: rifampicin docked to the wild-type protein. Figure S2: Post-docking analysis: 2D binding modes of RIF with the WT and mutated forms of *M. tuberculosis* rpoB. Molecular interactions were visualized using Discovery Studio Visualizer 4 (DS Visualizer) [72]. Figure S3: Post MD results: Preliminary assessment of the wild-type model runs 1 and 2. (A) The protein and ligand RMSDs represented as time evolution (i), kernel density estimation violin plots (ii), and frequency histograms. (B) The radius of gyration calculated for the entire protein, as well as residues within rifampicin binding pocket. Figure S4: 100ns protein RMSD evolution computed with respect to backbone atom positions. Color key: black: WT, red: mutants. Table S2: tabulated summary of average RMSD values acquired for each model. Figure S5: time-dependent RMSD evolution of rifampicin for each system. Color key: black: WT, red: mutant. Figure S6: Average per residue RMS fluctuation of rpoB computed based on C-α atom positions. Ligand binding regions are shaded grey. Blue bar indicates the position of associated mutation. Color code: black: WT, red: mutants. Figure S7: Structural mapping of the average per residue RMSF (calculated across all models). The color ranges from blue (low RMSF values) to red (high RMSF values). Figure S8: Time (ns) dependent Rg evolution of rpoB computed based on backbone atom positions. Color code: black: WT, red: mutants. Table S3: The proportion of variance captured by the top four principal components (PC1, PC2, PC3, and PC4). Generally, the first four PCs accounted for >60% of the variance. Trace values obtained following the diagonalization of the covariance are also tabulated. Figure S9: 2D projections of PC1 and PC2 as a function of time. Figure S10: Porcupine plots displaying concerted atomic motion acquired during simulation. Arrows represent the general direction of dominant motion, whereas the porcupine length represents the magnitude of motion. Figure S11: Structural comparison of dominant protein conformers extracted from low energy minimas on the conformational landscape (see Figure 5): WT (C1) versus mutants (C3, C4, C5, C6, C8, and C9). Color Key: WT: deep salmon, mutants: green. Table S4: Structural comparison of dominant protein conformers: WT (conformer: C1) versus mutants. Figure S12: Time-dependent hydrogen bond numbers formed between rpoB and rifampicin during simulation. Figure S13: Protein–ligand interactions visualized using Discovery Studio Visualizer 4 (DS Visualizer) [72]. The representative structures were extracted from low energy minimas representing dominant protein conformations. Figure S14: Bar plots showing per residue contribution to the total binding free energy for each model. Shaded regions indicate areas located within the ligand binding pocket. Table S5: Tabulated summary of residues contributing substantially (> ± 3X*sd*) to the total binding free energy in each model. -ve indicates the negative binding free energy value, while +ve indicates that a positive energy value was recorded. Figure S15: Per residue average *L* plots. Table S6: Average *BC* pairwise Pearson’s correlation values computed among models. The WT–RIF input is computed from per residue average values of run 1 and run 2. Figure S16: Plots of per residue average *BC*. Table S7: Residues possessing large average *BC* values (peaks) with respect to the WT systems (native representation). Table S8: Tabulated summary of residues that yielded large changes in average *BC* (*ΔBC*) due to mutations. Residues that yielded high average *BC* values are shown in bold. Residues unique to each model are underlined. Figure S17: Ensemble averaged residue contact map of the WT and mutated models. Supplementary_file S1: *rpoB* mutations retrieved from MUBII-TB-DB database. Supplementary_file S2: *rpoB* mutations prioritized in this study.

## Sample Availability

Not available.
