# Supplementary material for: The Structural Basis of Mycobacterium tuberculosis RpoB Drug-Resistant Clinical Mutations on Rifampicin Drug Binding
Source: Molecules. 2022 Jan 28;27(3):885. doi: 10.3390/molecules27030885 (PMC8839920; doi:10.3390/molecules27030885)
Supplement: Supplementary file 1 [file molecules-27-00885-s001.zip › Amusengeri_etal__Molecules_Supplementary_Material_AA.pdf]

Supplementary Material

The structural basis of *Mycobacterium tuberculosis* RpoB drug resistant clinical mutations on rifampicin drug binding

*Arnold Amusengeri[1], Asif Ullah Khan [2], and Özlem Tastan Bishop [1]**

[1]Research Unit in Bioinformatics (RUBi), Department of Biochemistry and Microbiology, Rhodes University, Grahamstown, 6140, South Africa; g16a7782@campus.ru.ac.za, O.TastanBishop@ru.ac.za

[2]Department of Biochemistry, Abdul Wali Khan University Mardan (AWKUM), Mardan-23200, Pakistan; asif@awkum.edu.pk

*** Correspondence:**
Asifullah Khan
asif@awkum.edu.pk; Tel: +92-333-9278471
Özlem Tastan Bishop
o.tastanbishop@ru.ac.za; Tel.: +27-46-603-8072

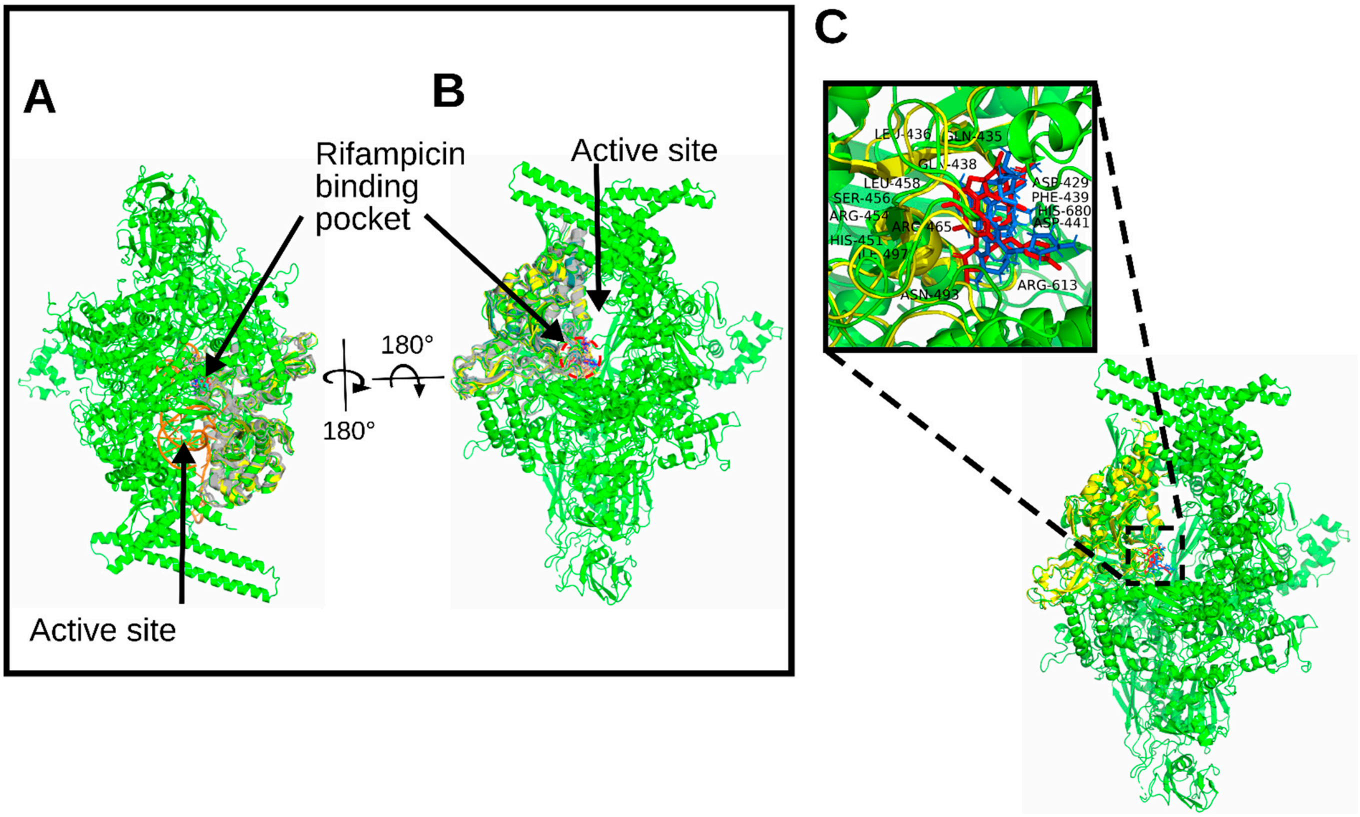

Figure S1. A: Graphical representation of the crystal structure of *Mycobacterium tuberculosis* transcription initiation complex, PDB ID: 5UHC [1], containing 3nt RNA in complex with rifampicin. The structure is superimposed to the wildtype and mutated models. Color code: Green: multiunit RNA polymerase complex; Brown: 3nt RNA strand; Yellow: Modeled wildtype structure of the β-subunit; Grey: Modeled mutated structures of the β-subunit. Rifampicin ligands docked to the structures is shown in sticks. **B:** 5UHC structure without the RNA strand: Depiction of the rifampicin binding pocket adjacent to the active center [2,3]. **C:** Zoomed in image of the rifampicin binding pocket depicting all residues within 3.7 A distance from RIF atoms. Color code: Red: co-crystallized rifampicin bound to 5UHC; Blue: rifampicin docked to the wildtype protein.

Table S1. Docking scores, RMSD and post-docking interactions (hydrogen bonds) of rifampicin (RIF) in WT and mutated models of rpoB.

Mutations	RMSD (Å)	Docking scores (kcal/mol)	Interacting residues
Wild Type	0.000	–13.80	Gln438, Phe439, Arg454, Ser456, Arg465
Val 176 Phe-RIF	1.468	-8.3	Arg613, Ile497
Gln 415 Tyr-RIF	0.018	-10.4	Gln438, Arg454
Asn 419 His-RIF	1.599	-6.7	Arg454
Ile 421 Val-RIF	1.200	-6.8	Gln438
Leu 436 Pro-RIF	1.279	-8.3	No interactions
Met 440 Val-RIF	1.653	-6.7	No interactions
Asp 441 Gly-RIF	1.538	-6.9	Asn493
Asp 441 Phe-RIF	1.657	-5.0	Gln438
Asp 441 Tyr-RIF	1.572	-6.1	Tyr441, Arg613
Asp 441 Val-RIF	1.272	-6.3	No interactions
Gln 443 Asp-RIF	1.432	-7.7	Asp441, Gln438
Asn 444 Thr-RIF	1.762	-5.4	Asn493, Arg465
Gly 448 Arg-RIF	1.725	-5.2	Asn493
Leu 449 Met-RIF	1.587	-6.8	No interactions
His 451 Gly-RIF	1.734	-5.0	Asn493
His 451 Pro-RIF	1.570	-6.5	Arg454, Asn493
His 451 Tyr-RIF	0.025	-10.0	Arg454
Arg 454 Gln-RIF	1.555	-6.1	Gln438
Ser 456 Gln-RIF	1.370	-6.0	Thr433, Arg465
Ser 456 Trp-RIF	1.829	-5.4	Asp441, Asn443, Arg454
Ala 457 Asp-RIF	1.571	-6.1	Ile497
Leu 458 Pro-RIF	0.022	-10.9	Arg 465
Pro 460 His-RIF	1.384	-7.6	Phe439
Gly 461 Asp-RIF	1.841	-5.0	No interactions
Leu 463 Phe-RIF	0.019	-9.3	Arg613
Arg 467 His-RIF	1.391	-9.0	Gln435, Gln438
Pro 489 Ser-RIF	0.027	-10.2	Phe439, Arg454
Ile 497 Phe-RIF	1.540	-7.6	Thr450
Arg 517 Cys-RIF	1.390	-7.6	Arg465
Val 519 Asp-RIF	1.469	-8.9	Asn493

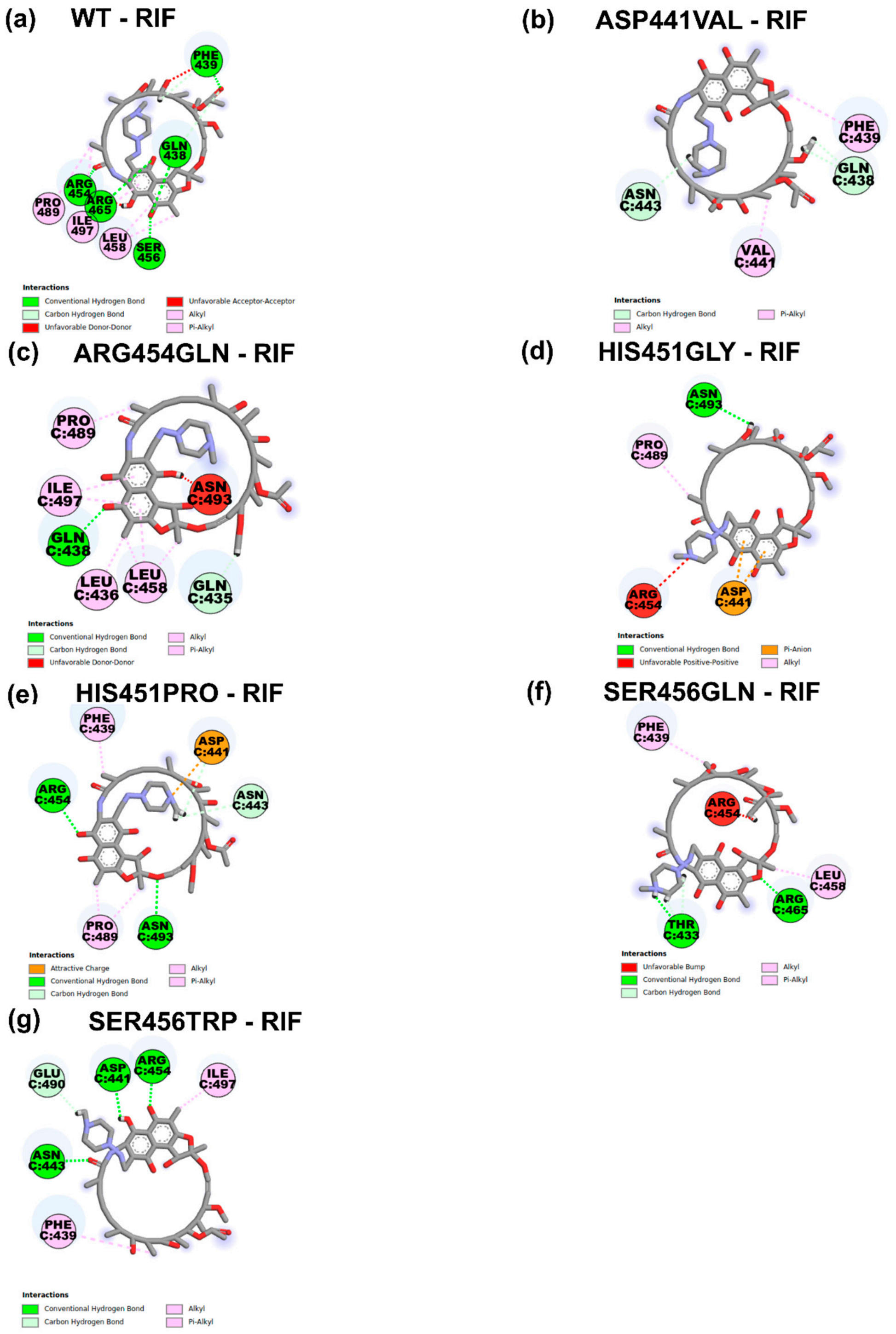

Figure S2. Post-docking analysis: 2D binding modes of RIF with the WT and mutated forms of *M. tuberculosis rpoB*. Molecular interactions were visualized using Discovery Studio Visualizer 4 (DS Visualizer) [4].

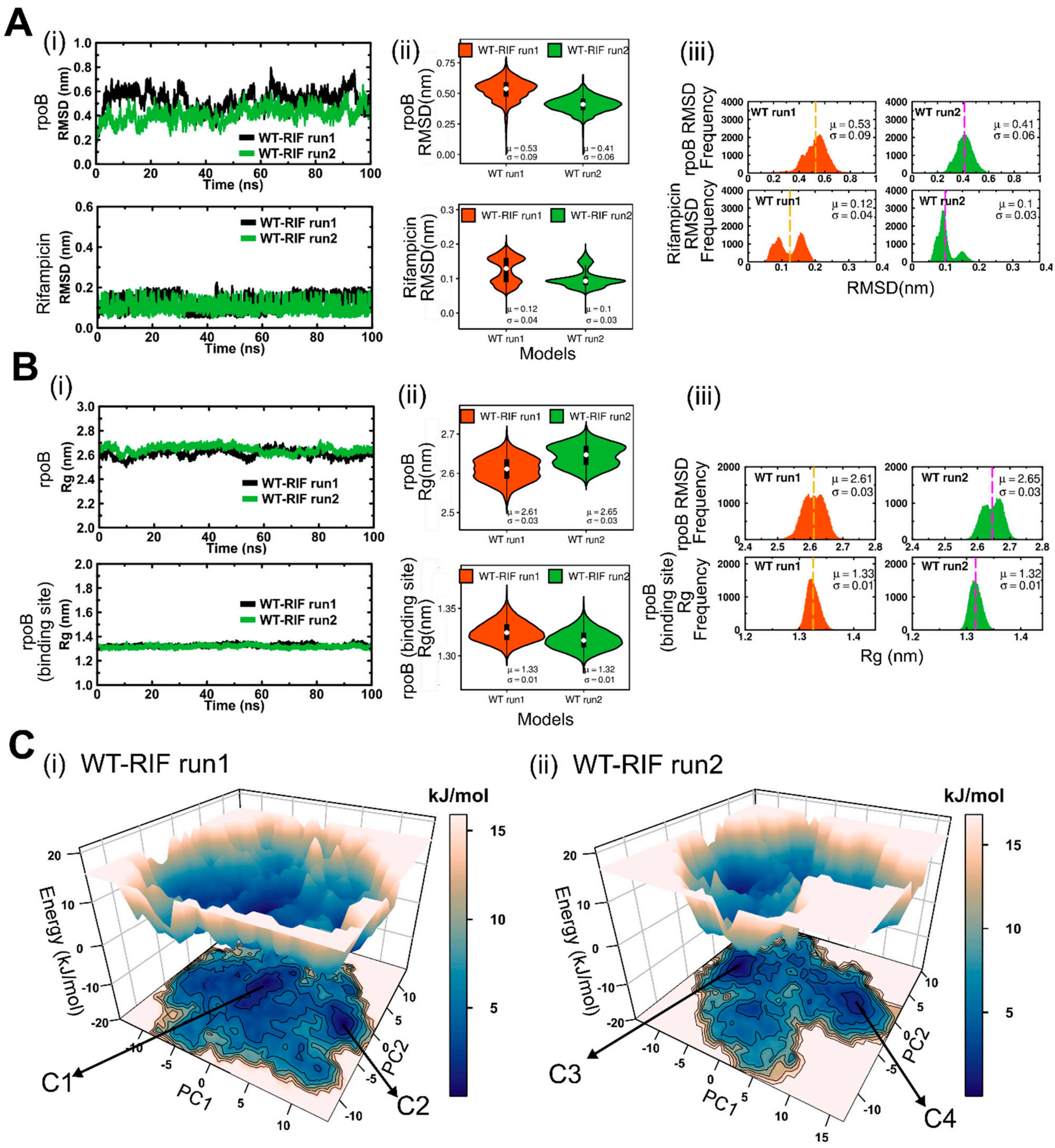

Figure S3. Post MD results: Preliminary assessment of the wild type model runs 1 and 2. (**A**) The protein and ligand RMSDs represented as time evolution (i), kernel density estimation violin plots (ii), and frequency histograms. (**B**) The radius of gyration calculated for the entire protein, as well as residues within rifampicin binding pocket. Colour key: Red: Run1, green: Run2. Yellow and magenta dashed lines in A(iii) and B(iii) highlight mean values of associated data. (**C**) Free energy landscapes computed as a function of PC1 and PC2. The colour ranges from white (maxima) to blue (minima). Identified conformers are labeled C1 to C4.

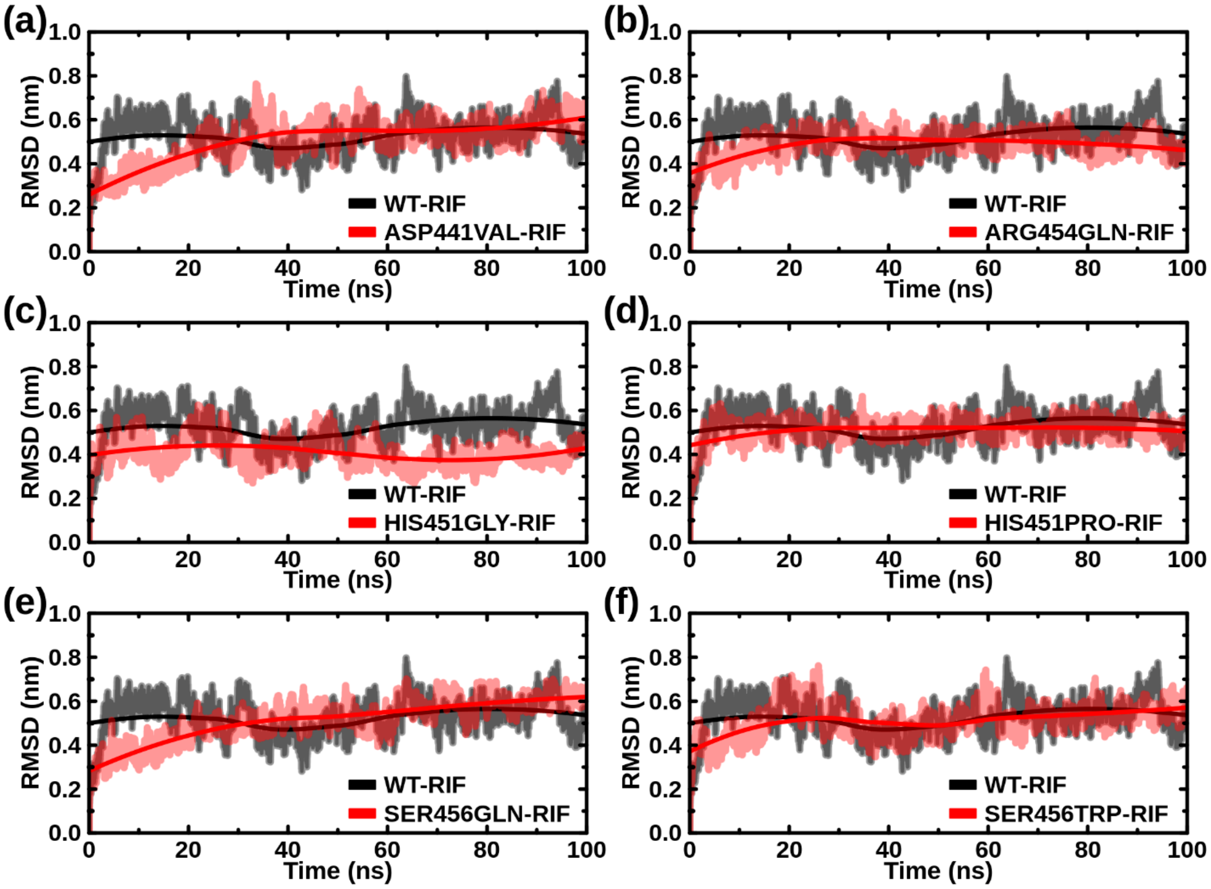

Figure S4. 100ns protein RMSD evolution computed with respect to backbone atom positions. Color key: Black: WT, Red: Mutants.

Table S2. Tabulated summary of average RMSD values acquired for each model.

System	Average RMSD
WT-RIF	0.5292
Asp441Val-RIF	0.5083
Arg454Gln-RIF	0.4862
His451Gly-RIF	0.4087
His451Pro-RIF	0.5129
Ser456Gln-RIF	0.5152
Ser456Trp-RIF	0.5137

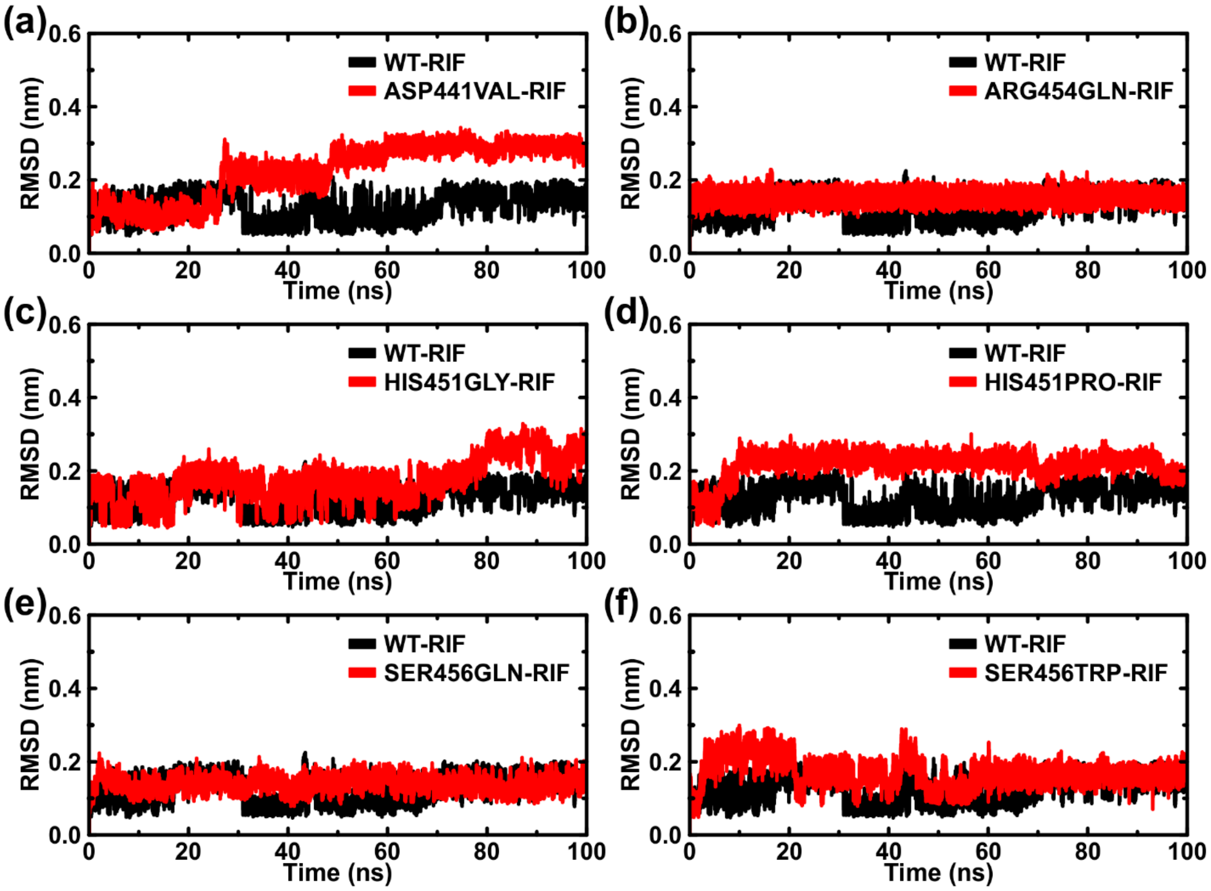

Figure S5: Time dependent RMSD evolution of rifampicin for each system. Color key: Black: WT, Red: Mutant.

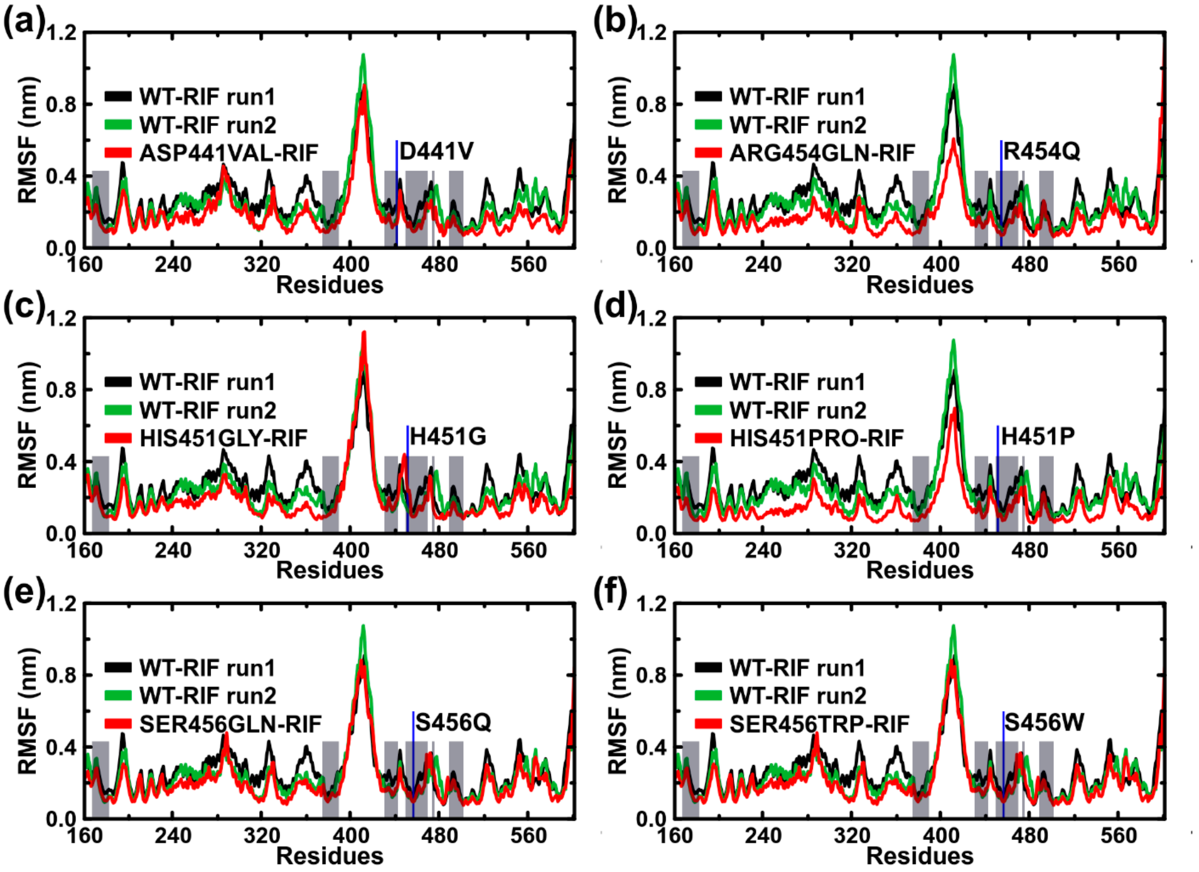

Figure S6. Average per residue RMS fluctuation of rpoB computed based on C-α atom positions. Ligand binding regions are shaded grey. Blue bar indicates the position of associated mutation. Color code: Black: WT, Red: Mutants.

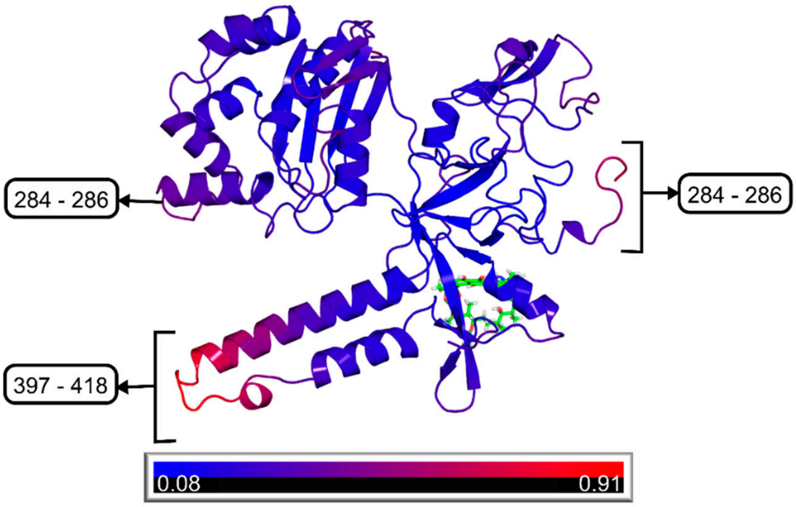

Figure S7. Structural mapping of the average per residue RMSF (calculated across all models). The color ranges from blue (low RMSF values) to red (high RMSF values).

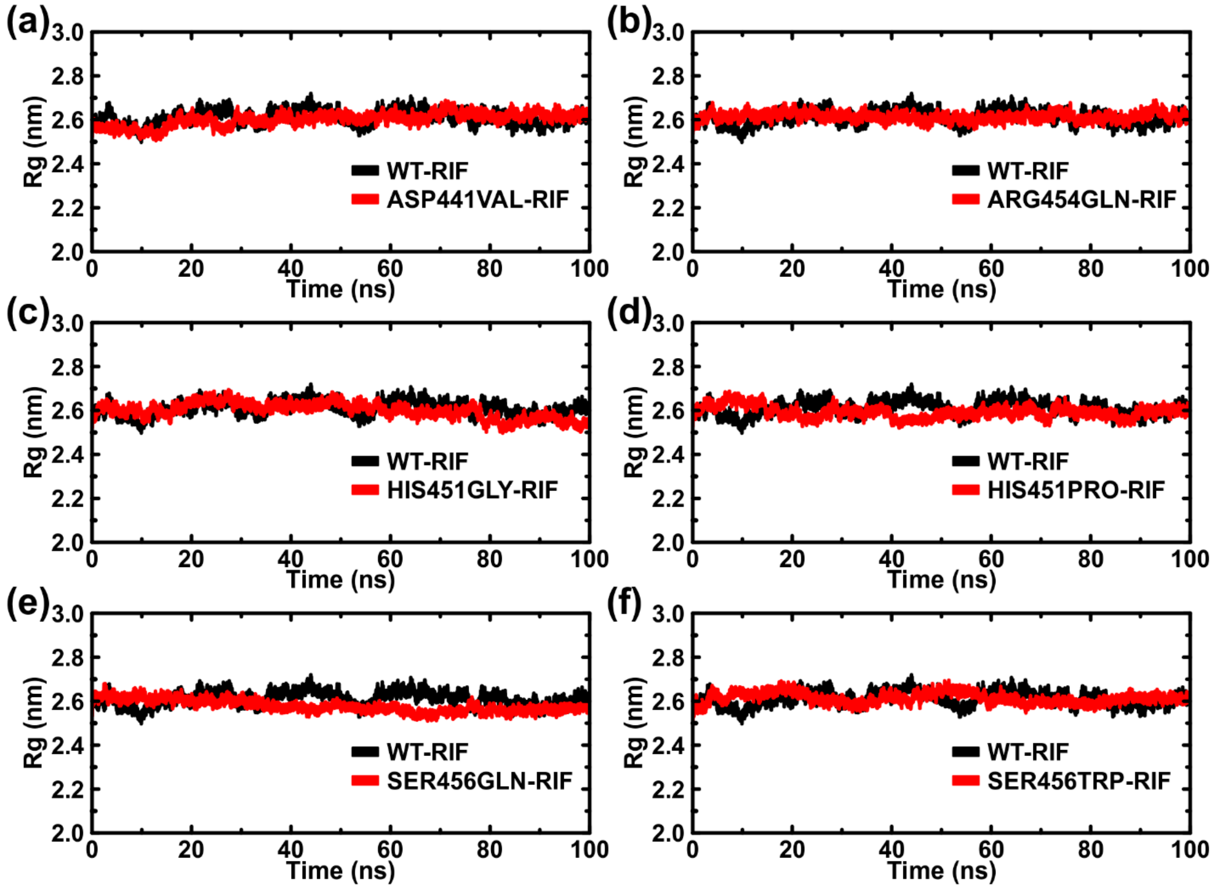

Figure S8. Time (ns) dependent Rg evolution of rpoB computed based on backbone atom positions. Color code: Black: WT, Red: Mutants.

Table S3. The proportion of variance captured by the top four principal components (PC1, PC2, PC3 and PC4). Generally, the first four PCs accounted for > 60% of the variance. Trace values obtained following the diagonalization of the covariance are also tabulated.

System	Percentage variance				Trace values
	PC1	PC2	PC3	PC4	(Sum of 3969 eigenvalues)
WT-RIF run1	34.71 %	18.04 %	14.92 %	9.39 %	113.69
WT-RIF run2	56.96%	14.14%	6.37%	4.15%	101.87
Asp441Val-RIF	42.66 %	19.90 %	6.01 %	4.44 %	65.73
Arg454Gln-RIF	25.78 %	21.81 %	12.72 %	8.43 %	53.87
His451Gly-RIF	49.20 %	20.86 %	5.54 %	3.52 %	78.94
His451Pro-RIF	22.59 %	17.66 %	12.20 %	6.85 %	39.58
Ser456Gln-RIF	54.46 %	13.73 %	6.24 %	3.88 %	82.58
Ser456Trp-RIF	38.75 %	19.46 %	14.54 %	4.52 %	77.86

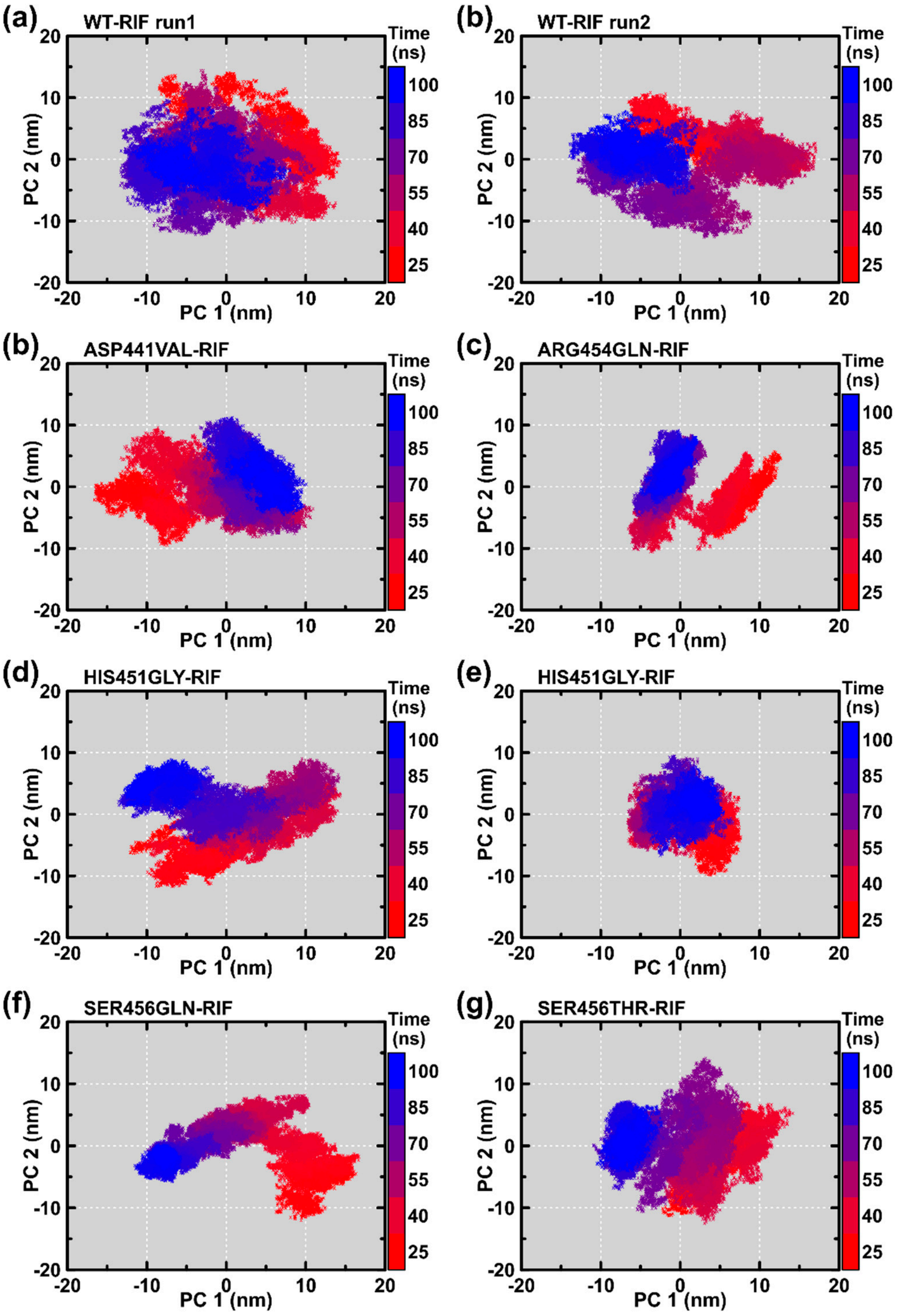

Figure S9. 2D projections of PC1 and PC2 as a function of time.

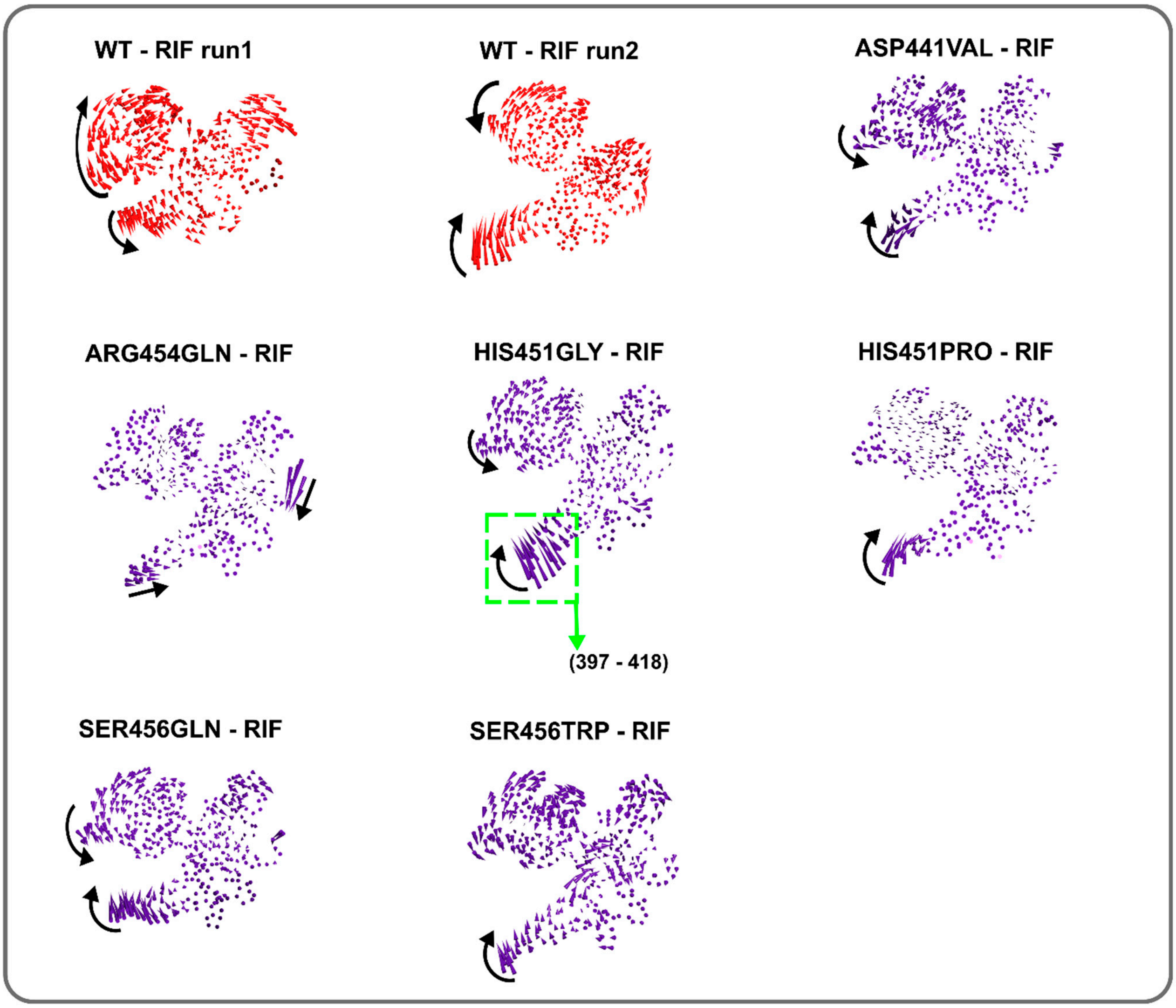

Figure S10. Porcupine plots displaying concerted atomic motion acquired during simulation. Arrows represent the general direction of dominant motion whereas the porcupine length represents the magnitude of motion.

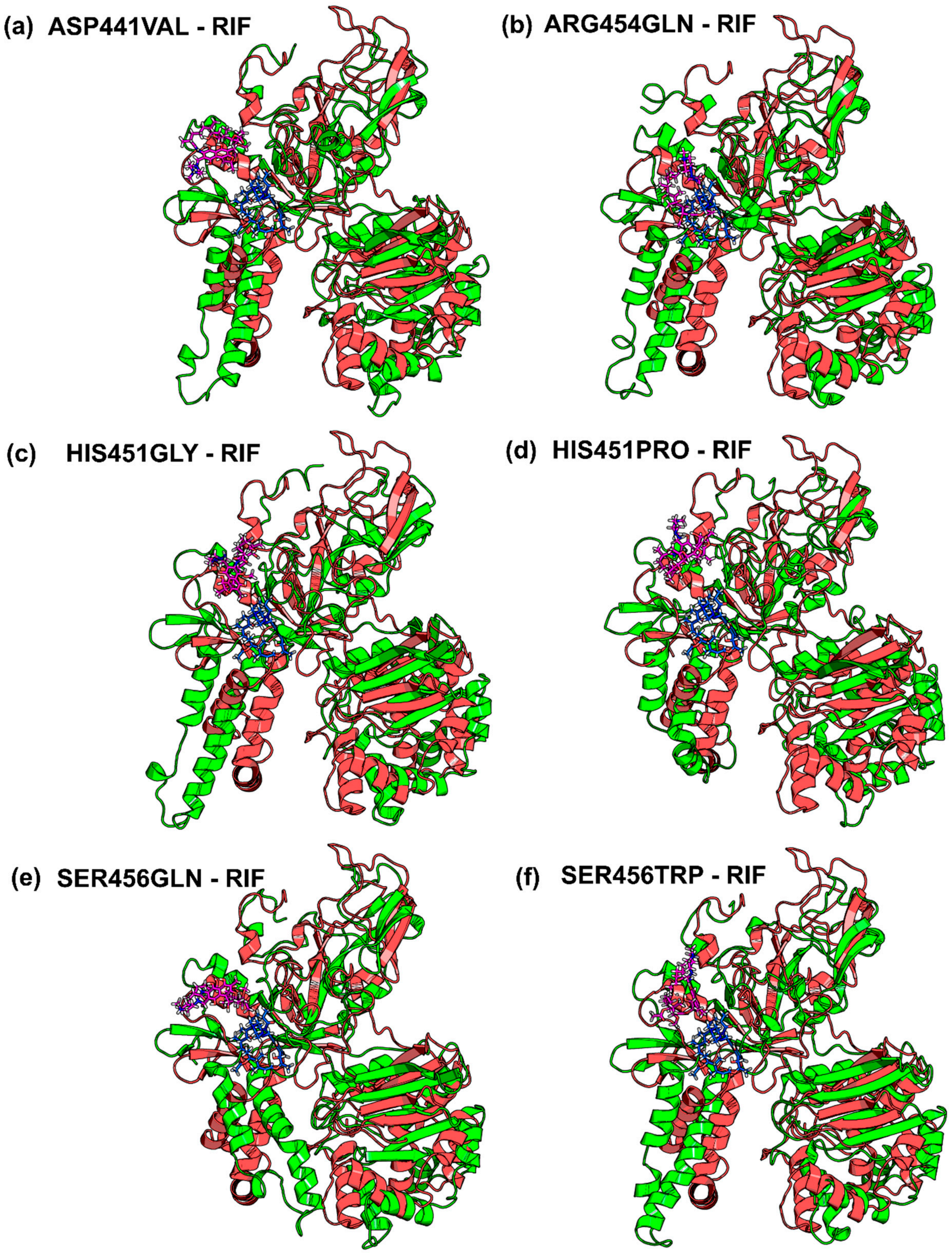

Figure S11. Structural comparison of dominant protein conformers extracted from low energy minimas on the conformational landscape (**See Figure 5**): WT (C1) versus mutants (C3, C4, C5, C6, C8, C9). Colour Key: WT: deep salmon, Mutants: green.

Table S4. Structural comparison of dominant protein conformers: WT (conformer: C1) versus mutants.

Protein system	Dominant Conformer	RMSDs (nm)
arg454gln	C3	0.8010
asp441val	C4	0.8270
his451gly	C5	0.8455
his451pro1	C6	0.9042
ser456gln	C8	0.8937
ser456trp	C9	0.8081

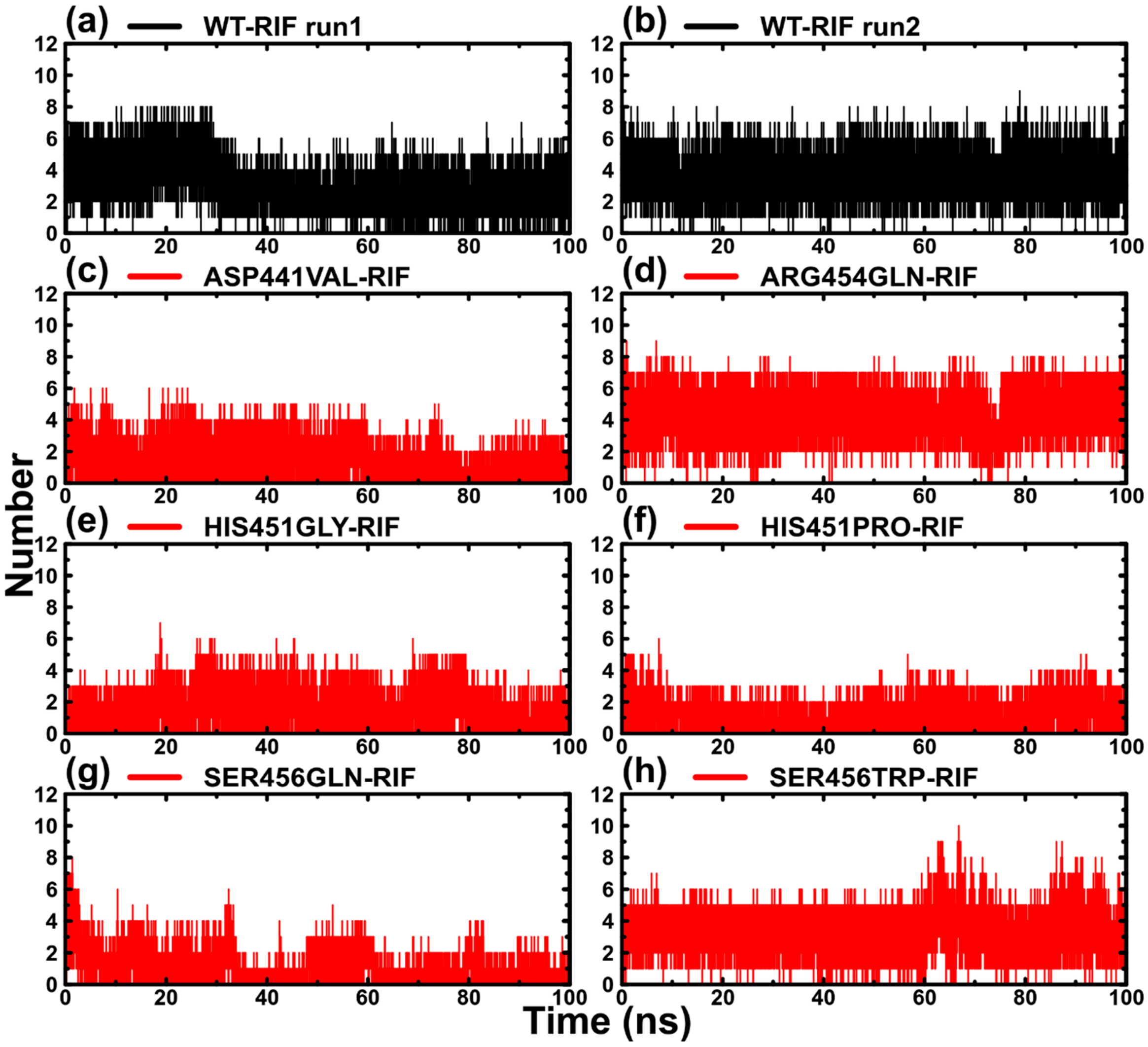

Figure S12. Time-dependent hydrogen bond numbers formed between rpoB and rifampicin during simulation.

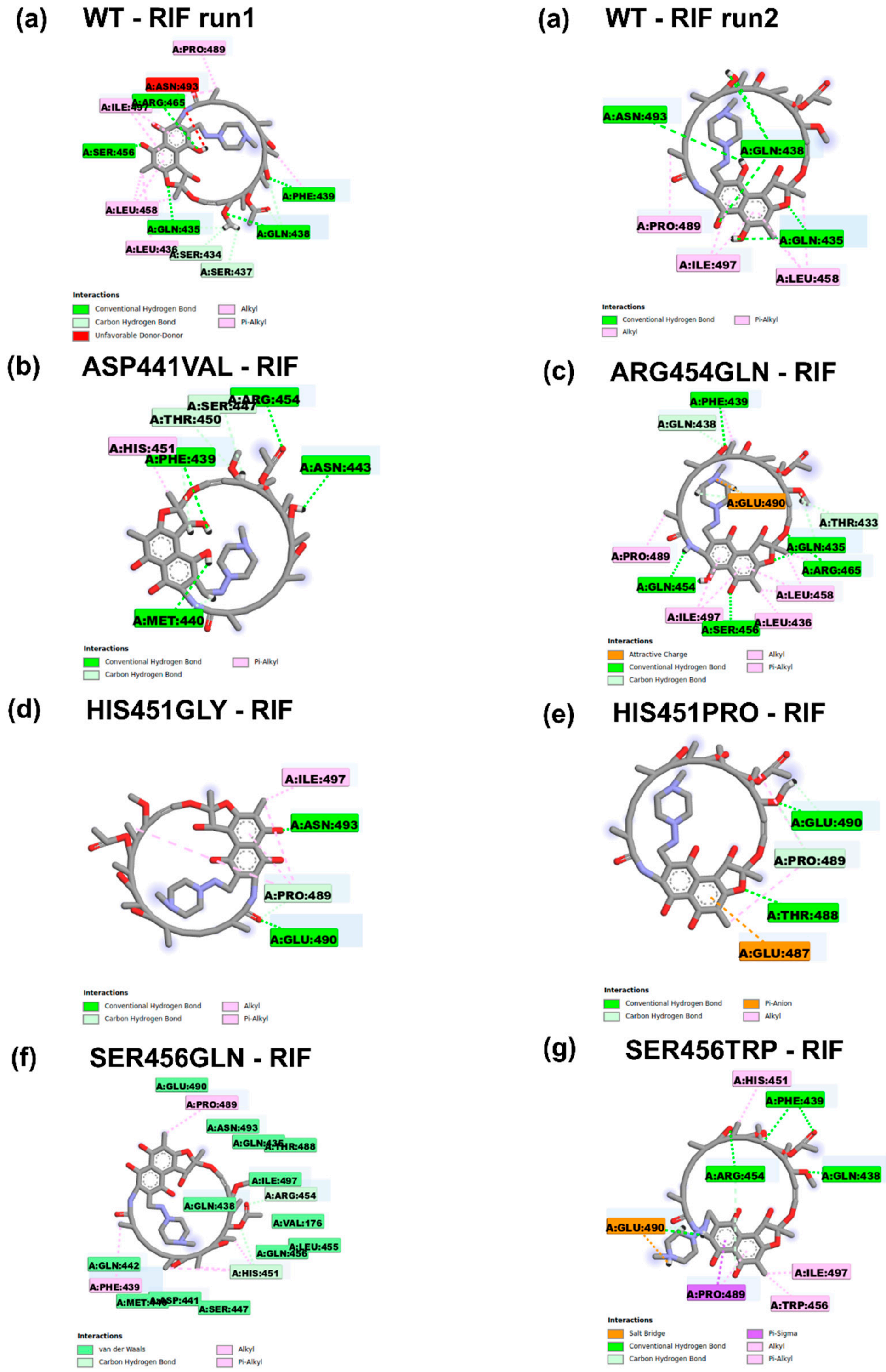

Figure S13. Protein-ligand interactions visualized using Discovery Studio Visualizer 4 (DS Visualizer) [4] . The representative structures were extracted from low energy minimas representing dominant protein conformations.

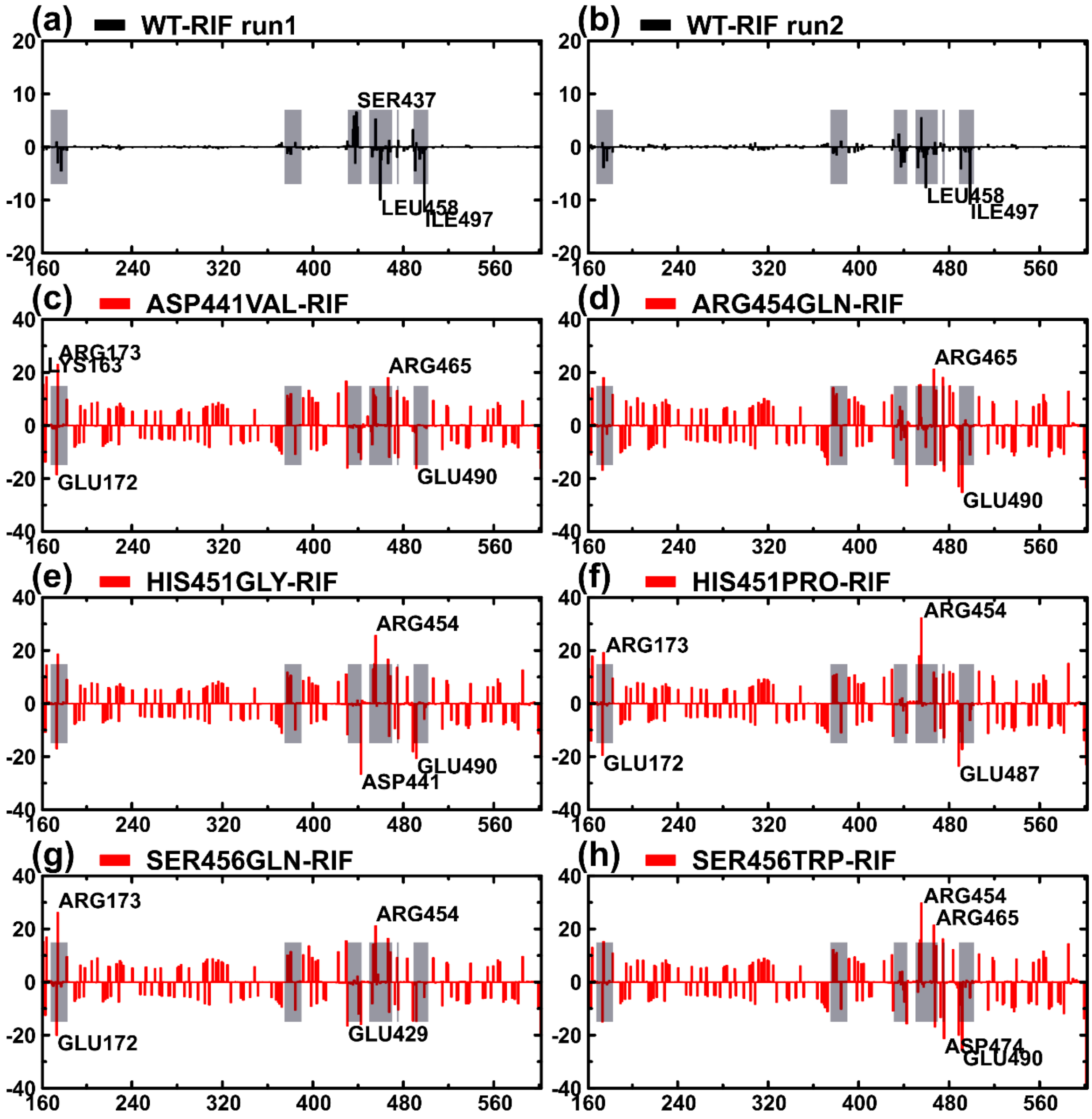

Figure S14. Bar plots showing per residue contribution to the total binding free energy for each model. Shaded regions indicate areas located within the ligand binding pocket.

Table S5. Tabulated summary of residues contributing substantially (> +/- 3X*sd*) to the total binding free energy in each model. -ve indicates that negative binding free energy value, while +ve indicates that a positive energy value was recorded.

Protein	Energy value (+ve: positive, -ve: negative)	Residue
WT-RIF run1	+ve	SER434, GLN435, SER437, GLN438, ARG454, GLU487
	-ve	VAL176, LEU458, PRO489, ILE497
WT-RIF run2	+ve	ARG454
	-ve	ARG173, LEU436, GLN438, PHE439, HIS451, LEU458, PRO489, ILE497
Arg441Val-RIF	+ve	MET160, LYS163, ARG173, LYS428, ARG465
	-ve	ASP600, GLU490, GLU429, GLU172
His451Pro-RIF	+ve	LYS163, ARG173, LYS452, ARG453, ARG454
	-ve	GLU172, GLU487, GLU490, ASP600
His451Gly-RIF	+ve	ARG173, ARG454, ARG465
	-ve	GLU172, ASP441, GLU487, GLU490, ASP600
Arg454Gln-RIF	+ve	ARG173, ARG465, ARG473
	-ve	ASP441, ASP474, GLU487, GLU490, ASP600
Ser456Gln-RIF	+ve	MET160, LYS163, ARG173, LYS428, ARG454, ARG465
	-ve	GLU172, GLU429, ASP441, ASP600
Ser456Trp-RIF	+ve	ARG454, ARG465
	-ve	GLU466, ASP474, GLU487, GLU490, ASP600

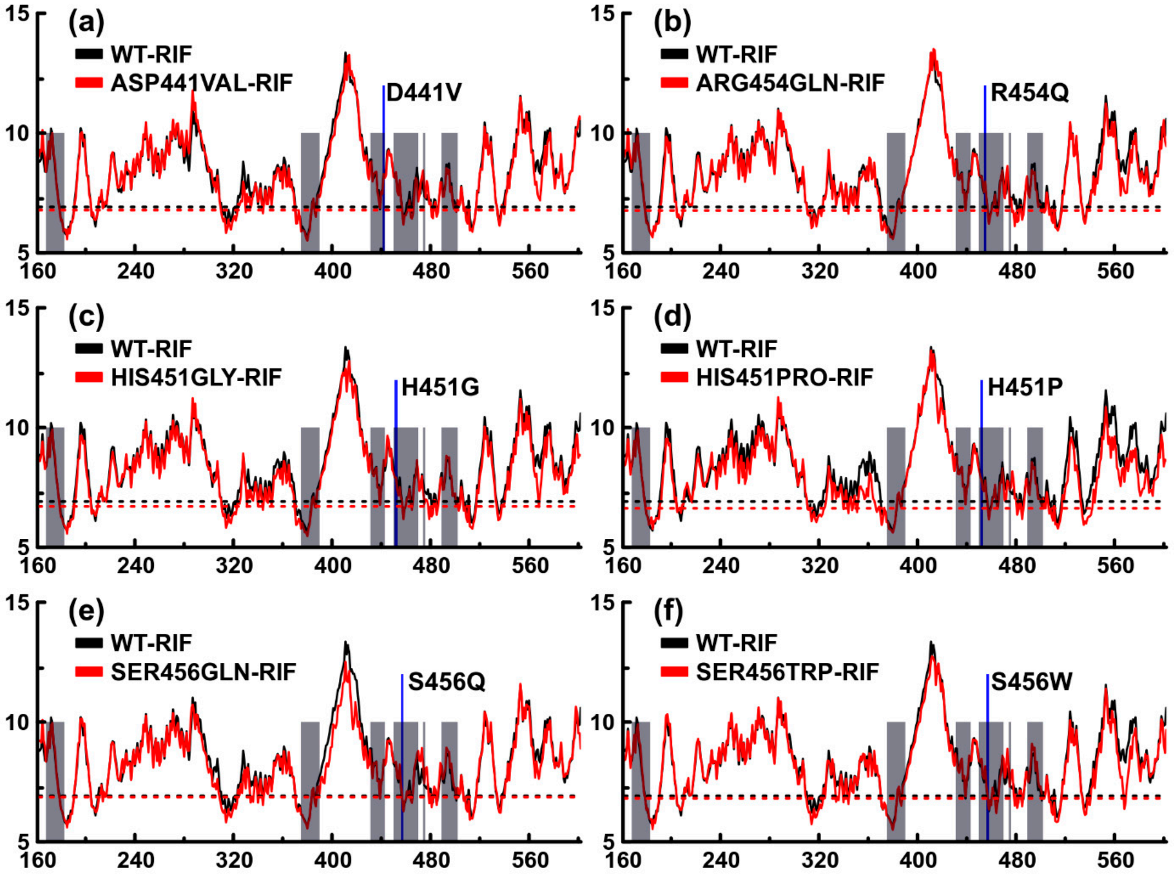

Figure S15. Per residue Average *L* plots. The WT-RIF highlights the average value from run1 and run2. Colour key: Black: Wildtype, Red: Mutants, Grey: residues within ligand binding region. Dashed lines indicate threshold values (1xSD from the mean) used to identify low average *L* regions (dips); WT-RIF: 6.91, Asp441Val-RIF: 6.79, Arg454Gln-RIF:6.76, His451Gly-RIF: 6.71, His451Pro-RIF: 6.63, Ser456Gln-RIF: 6.87, Ser456Trp-RIF: 6.82.

Table S6. Average *BC* pairwise Pearson's correlation values computed among models. The WT-RIF input is computed from per residue average values of run1 and run2.

System	WT-RIF	Asp441Val-RIF	Arg454Gln-RIF	His451Gly-RIF	His451Pro-RIF	Ser456Gln-RIF	Ser456Trp-RIF
WT-RIF	1						
Asp441Val-RIF	0.87	1					
Arg454Gln-RIF	0.88	0.87	1				
His451Gly-RIF	0.89	0.91	0.94	1			
His451Pro-RIF	0.83	0.84	0.85	0.90	1		
Ser456Gln-RIF	0.88	0.87	0.83	0.87	0.80	1	
Ser456Trp-RIF	0.86	0.94	0.84	0.89	0.82	0.87	1

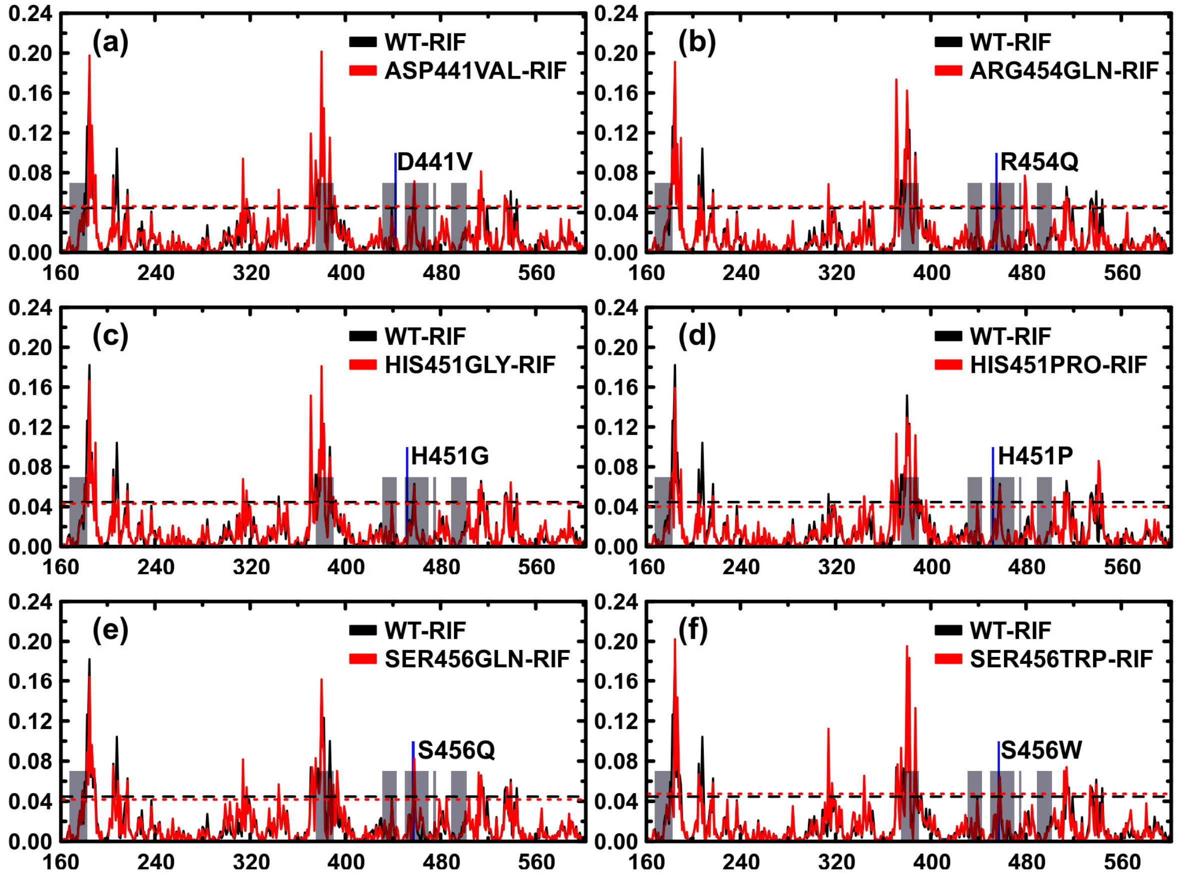

Figure S16. Plots of per residue average *BC*. WT-RIF highlights the average value from run1 and run2. Color code: Black: WT, Red: Mutants. Grey: residues within ligand binding region. Dashed lines indicate threshold values (2X*sd*) used to identify low average *L* regions (dips); WT-RIF: 0.045, Asp441Val-RIF: 0.046, Arg454Gln-RIF: 0.046, His451Gly-RIF: 0.043, His451Pro-RIF: 0.040, Ser456Gln-RIF: 0.042, Ser456Trp-RIF: 0.047.

Table S7. Residues possessing large average *BC* values (peaks) with respect to the WT systems (native representation).

Model	Residues
WT-RIF	LEU179-TYR186, ASP188, LYS203-SER207, ASP215, ASN317, ILE342, ASP369-ILE370, HIS372-THR380, LEU384-ASN387, ARG390, SER394, SER456, ILE512-THR514, ALA533, ASP537, ALA542

Table S8. Tabulated summary of residues that yielded large changes in average *BC* (Δ BC) due to mutations. Residues that yielded high average *BC* values are shown in bold. Residues unique to each model are underlined.

Models	Change	Residues
ASP441VAL	Positive	**LEU179, ARG181, SER182, VAL204, PRO206,** GLU213, VAL381, **GLN386**
	Negative	**VAL185**, GLY312, LYS315, **LEU378, THR380**, **ILE385,** ILE389, GLY510
SER456TRP	Positive	**ARG181, SER182, VAL204, PRO206, PHE373, GLY374, ASN375, ARG377**
	Negative	ASP188, GLY312, **ASP369, ILE370, ARG376, LEU378, ARG379**, PRO477, SER478, GLY510
SER456GLN	Positive	**ARG181, SER182, VAL204, PRO206, GLY374, ASN375, ARG377**, GLN386
	Negative	**ASP188,** GLY312, LYS315, **ASP369, ARG376, LEU378, THR380**, GLY510
ARG454GLN	Positive	**ARG181, SER182, VAL204, PRO206, PHE373, GLY374, ASN375, ARG377**
	Negative	VAL365, GLU366, **ARG376, ILE385**, GLY510, HIS539, VAL540
HIS451GLY	Positive	**ARG181, SER182, ASP188, PRO206**, GLU213, **ASP369, HIS372, ASN375, ARG377**, VAL381, **GLN386, ASN387, ARG390**
	Negative	PHE187, GLY209, GLY312, LYS315, ASP371, **LEU378,** GLN388, VAL391, GLY392, GLY461, GLY510
HIS451PRO	Positive	**ARG181, SER182, ASP188**, **PRO206, ASP369, HIS372, ASN375**
	Negative	**VAL185,** GLY312, LYS315, **LEU378, THR380**, ILE385, GLY510

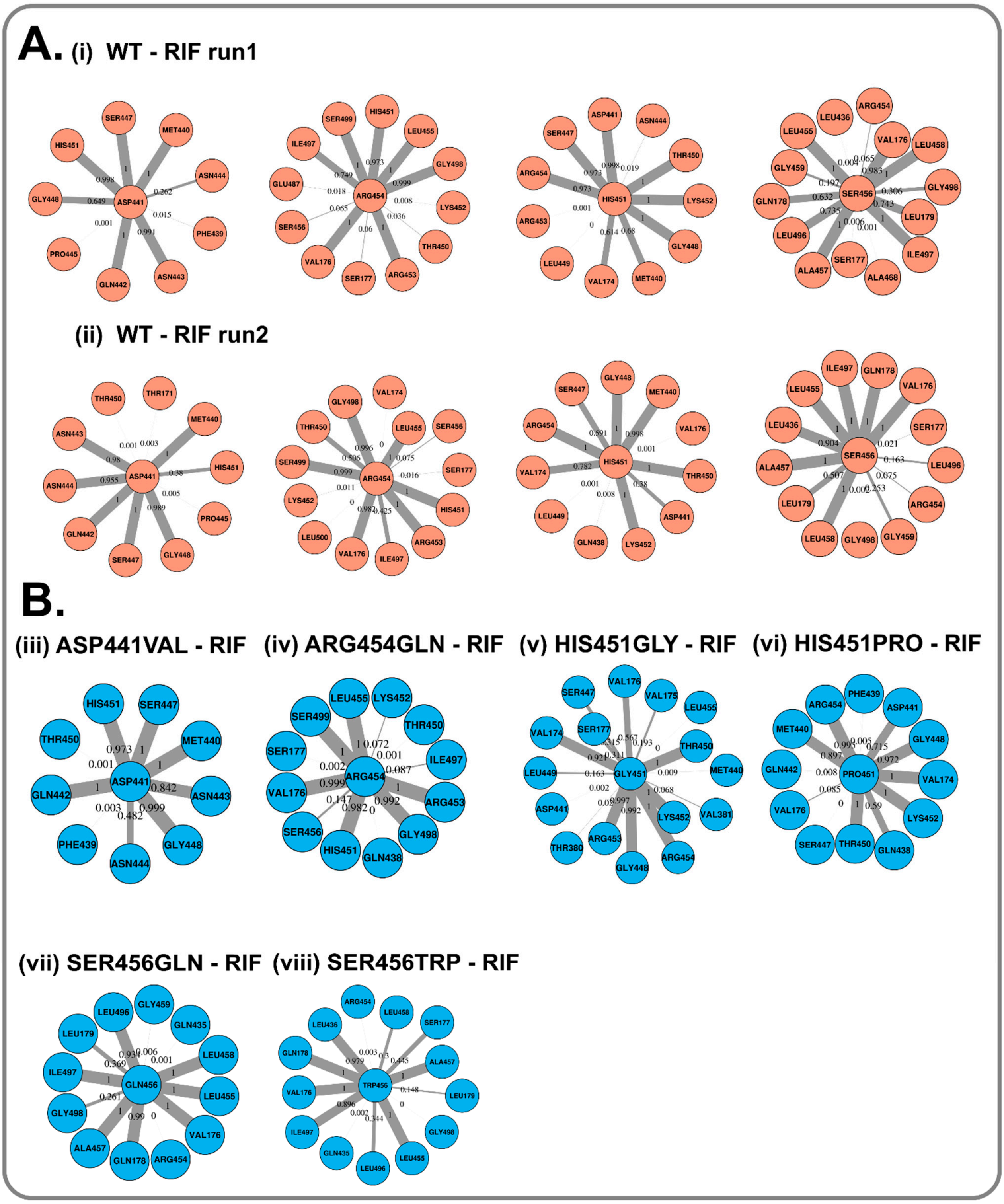

Figure S17. Ensemble averaged residue contact map of the WT and mutated models.

Supplementary file S1. RpoB mutations retrieved from MUBII-TB-DB database.

Supplementary file S2: RpoB mutations prioritized in this study.

REFERENCES

1. Lin, W.; Mandal, S.; Degen, D.; Liu, Y.; Ebright, Y.W.; Li, S.; Feng, Y.; Zhang, Y.; Mandal, S.; Jiang, Y.; et al. Structural Basis of Mycobacterium tuberculosis Transcription and Transcription Inhibition. *Mol. Cell* **2017**, doi:10.1016/j.molcel.2017.03.001.

2. Molodtsov, V.; Scharf, N.T.; Stefan, M.A.; Garcia, G.A.; Murakami, K.S. Structural basis for rifamycin resistance of bacterial RNA polymerase by the three most clinically important RpoB mutations found in Mycobacterium tuberculosis. *Mol. Microbiol.* **2017**, doi:10.1111/mmi.13606.

3. Campbell, E.A.; Korzheva, N.; Mustaev, A.; Murakami, K.; Nair, S.; Goldfarb, A.; Darst, S.A. Structural Mechanism for Rifampicin Inhibition of Bacterial RNA Polymerase. *Cell* **2001**, *104*, 901–912, doi:10.1016/S0092-8674(01)00286-0.

4. BIOVA BIOVIA Discovery Studio | Predictive Modeling & Science Simulation Software App Available online: http://accelrys.com/products/collaborative-science/biovia-discovery-studio/ (accessed on May 27, 2016).
